# Exploring Bioactive Components and Assessing Antioxidant and Antibacterial Activities in Five Seaweed Extracts from the Northeastern Coast of Algeria

**DOI:** 10.3390/md22060273

**Published:** 2024-06-12

**Authors:** Nawal Bouzenad, Nesrine Ammouchi, Nadjla Chaib, Mohammed Messaoudi, Walid Bousabaa, Chawki Bensouici, Barbara Sawicka, Maria Atanassova, Sheikh F. Ahmad, Wafa Zahnit

**Affiliations:** 1 Department of Process Engineering, Faculty of Technology, University 20 August 1955, Skikda 21000, Algeria; 2 Laboratory of Interactions, Biodiversity, Ecosystems and Biotechnology (LIBEB), University 20 August 1955, Skikda 21000, Algeria; 3 Department of Sciences and Technology, Faculty of Technology, University 20 August 1955, Skikda 21000, Algeria; ammouchi3@gmail.com; 4 Laboratoire de Recherche sur la Physico-Chimie des Surfaces et Interfaces (LRPCSI), University 20 August 1955, Skikda 21000, Algeria; 5 Laboratory of Catalysis, Bioprocesses and Environment (LCBE), University 20 August 1955, Skikda 21000, Algeria; 6 Nuclear Research Centre of Birine, Djelfa 17200, Algeria; messaoudi2006@yahoo.fr; 7 Scientific and Technical Research Center in Physico-Chemical Analysis (CRAPC), BP384, Bou-Ismail 42004, Algeria; bousabaa7@gamil.com; 8 Laboratory of Biochemistry, Biotechnology and Health Division, Center for Research in Biotechnology, Constantine 25000, Algeria; chawkiislam@yahoo.fr; 9 Department of Plant Production Technology and Commoditties Science, University of Life Sciences in Lublin, Akademicka 15 Str., 20-950 Lublin, Poland; barbara.sawicka@up.lublin.pl; 10 Scientific Consulting, Chemical Engineering, University of Chemical Technology and Metallurgy, 1734 Sofia, Bulgaria; atanassovamarias@uctm.edu; 11 Department of Pharmacology and Toxicology, College of Pharmacy, King Saud University, Riyadh 11451, Saudi Arabia; 12 Laboratory of Valorization and Promotion of Saharan Resource (VPRS), Faculty of Mathematics and Matter Sciences, University of Ouargla, Road of Ghardaia, Ouargla 30000, Algeria

**Keywords:** antibacterial activity, antioxidant activity, bioactive compounds, elementary analysis, nutritional and phytochemical composition, seaweed

## Abstract

The main goal of this study was to assess the bioactive and polysaccharide compositions, along with the antioxidant and antibacterial potentials, of five seaweeds collected from the northeastern coast of Algeria. Through Fourier transform infrared spectroscopy analysis and X-ray fluorescence spectroscopy, the study investigated the elemental composition of these seaweeds and their chemical structure. In addition, this study compared and identified the biochemical makeup of the collected seaweed by using cutting-edge methods like tandem mass spectrometry and ultra-high-performance liquid chromatography, and it searched for new sources of nutritionally valuable compounds. According to the study’s findings, *Sargassum muticum* contains the highest levels of extractable bioactive compounds, showing a phenolic compound content of 235.67 ± 1.13 µg GAE·mg^−1^ and a total sugar content of 46.43 ± 0.12% DW. Both *S. muticum* and *Dictyota dichotoma* have high concentrations of good polyphenols, such as vanillin and chrysin. Another characteristic that sets brown algae apart is their composition. It showed that *Cladophora laetevirens* has an extracted bioactive compound content of 12.07% and a high capacity to scavenge ABTS^+^ radicals with a value of 78.65 ± 0.96 µg·mL^−1^, indicating high antioxidant activity. In terms of antibacterial activity, *S. muticum* seaweed showed excellent growth inhibition. In conclusion, all five species of seaweed under investigation exhibited unique strengths, highlighting the variety of advantageous characteristics of these seaweeds, especially *S. muticum*.

## 1. Introduction

Seaweed is a varied group of about 15,000 species with a long history of use in numerous civilizations worldwide. They are particularly valued in Asia for their roles in medicine and cooking, where they are used with caution [1]. Seaweed has recently gained considerable interest from a range of industries, including biomedicine, due to their potential industrial possibilities in cosmetics, food, pharmaceuticals, and textiles [2,3]. In addition to their traditional use as anti-ulcerates, laxatives, and antibiotics due to their medicinal components [4]. The diet of indigenous coastal communities includes seaweed because of its high concentration of vital proteins, vitamins, and minerals [5].

The pharmaceutical industry uses secondary metabolites produced by seaweed, making them an important and versatile marine resource [6]. Seaweeds come in a variety of colors, and all of them contain a high amount of naturally occurring bioactive chemicals that have a variety of biological effects [7]. These include antioxidant, anticancer [8], antibacterial, and antifungal properties [9]. Carotenoids, antioxidant enzymes, polyphenols, ascorbic acid, tocopherols, and other antioxidant molecules all play a part in the antioxidant activity of seaweed [10]. It is also highly appreciated for its nutritional properties rich in minerals (calcium, iron, copper, and iodine; polysaccharides such as agaragar, alginate, and carrageenan; complete proteins with all essential amino acids; and lipids, especially polyunsaturated fatty acids; etc.) [11,12,13]. Seaweed is also rich in micronutrients such as vitamins A, B_1_, B_12_, C, D, and E [14], as well as flavonoids, phlorotanins, bromophenols, phenolic terpenoids, and additional phenolic compounds. Furthermore, seaweed’s fat- and water-soluble vitamins have been shown to lessen the incidence of thrombosis, atherosclerosis, and heart disease [15,16]. Free radicals can be neutralized by the phenolic and carotenoid compounds in seaweed, which helps to slow down oxidative deterioration and prevent degenerative diseases [17]. The increased interest in using seaweed is mainly due to its great potential as a source of bioactive substances that can be used in the food, pharmaceutical, and medical industries, as well as in many other industrial applications. Seaweed species abound along Algeria’s 1200 km southern Mediterranean coastline, which is rich in algae varieties. Numerous research projects have been conducted to investigate this rich algal diversity, as *Cystoseira compressa*, *Ericaria mediterranea* (formerly *Cystoseira mediterranea*), *Halopteris scoparia*, *Dictyota fasciola*, *Padina pavonica* (Paheophyceae), *Ellisolandia elongate Corallina elongate* (Rhodophyta), and *Cladophora* sp. (Chlorophyta) inventoried in Ténès, northwest Algeria [18], *Corallina officinalis* (Rhodophyta) in the Gulf of Arzew (west coast of Algeria) [19], in the same region on Oran coasts *Asparagopsis taxiformis* and *Hypnea musciformis* (Rhodophyta) were studied [20], *Dictyopteris polypodioides (*formerly *Dictyopteris membranacea* in western Algeria at Tipaza [21], and *Sargassum muticum* at Sidi Fredj (central Algerian coast) [22]. In eastern Algeria, *E. mediterranea* was sampled on the coast of the wilaya of Bejaia [23]. This algal variety demonstrates Algeria’s ecological richness and diversity and the potential for new scientific discoveries and advancements.

The main goals of the present research were: (1) examination of the chemical and biochemical composition of five seaweed species collected in the Gulf of Stora in the Mediterranean Sea in northeastern Algeria by using multiple approaches; (2) examination and checking of their elemental content and chemical structure; and (3) evaluation of the bioactivity of crude methanol extracts, ethyl acetate fractions, and n-butanol fractions from these seaweed species. The primary focus was on their antioxidant and antibacterial properties. In addition, ultra-high performance liquid chromatography with electrospray ionization coupled with tandem mass spectrometry was used in this study to identify and compare the biochemical composition of the collected seaweed, as well as to investigate new sources of valuable compounds that can be extracted and purified from the sampled seaweed.

## 2. Results and Discussion

### 2.1. Extraction Efficiency of the Various Components

The yields of the bioactive compounds and polysaccharide extraction from the five seaweed collected samples are displayed in Table 1.

The extraction yields of bioactive compounds from the seaweeds in this study show significant variations and are ranked as follows, in descending order: *D. dichotoma* (27.07 ± 1.19%), *C. laetevirens* (12.07 ± 0.44%), *U. lactuca* (9.55 ± 0.12%), *C. officinalis* (6.11 ± 0.12%), and *S. muticum* (5.29 ± 0.44%).

The extraction yield of bioactive compounds from *D. dichotoma* was 27.07%. In contrast, the yield from the same species, gathered at the shores near Bahía Bustamante in summer (Chubut Province, Argentina) [24], amounted to only 3.6% for a methanolic crude extract. Conversely, *D. dichotoma*, hand-collected in December along the Kachchh coast in Gujarat, India [25], showcased a notably higher yield of 37.97% using the same solvent, underscoring the substantial variability in yield within this species. Another Mediterranean harvest of *D. dichotoma*, resulting in a 14.22% yield for a methanolic extract, occurred in May 2015 during a scuba dive in Bou Ismail Bay (central Algerian coast) [26].

Our investigation revealed that *S. muticum* contained 5.29% of extracted bioactive compounds. Conversely, various *Sargassum* species collected across different regions exhibited significantly variable yields. For example, ethanol absolute extraction of *S. aquifolium*, harvested along the Kuwaiti coast, manifested a higher yield of 11.9% [27]. In contrast, methanolic extraction of *S. oligocystum* yielded 7.11% in Malaysia [28], and methanol (1:10 *w*/*v*) extraction in Manado, Indonesia, resulted in a 4.95% yield [29], emphasizing the diversity of extraction yields within this genus.

The red seaweed *C. officinalis*, collected from our northeastern Algerian coast, exhibited an extraction yield of bioactive compounds of 6.11%, slightly surpassing that of the methanolic extract of *C. officinalis* (4.3%) collected in Egypt (Abu Qir Bay) [30].

The extraction of bioactive compounds from *U. lactuca* (9.55%) (Table 1) diverges from the results obtained for the same genus harvested in similar Mediterranean waters, such as the methanolic extract of *Ulva lactuca* (formerly *U. fasciata*) (15.0%) in Abu Qir Bay (Egypt) [30], while the methanolic extract of *U. intestinalis* harvested on the central Algerian coast (Bou Ismaïl Bay) demonstrates a comparable yield (10.55%) [26].

A total of 12.07% of the bioactive compounds in *C. laetevirens* were extracted from our northeastern Algerian coastline. This yield contrasts with other results from *Cladophora* collections, revealing significant variations in extraction yields. *Cladophora* sp. collected from the Kuwaiti coast displayed the highest extraction yield of bioactive compounds (26.5%) for the 50% ethanol extract [27], while *C. glomerata* from Thailand exhibited the lowest yield (3.82%) for the methanolic extract [31].

In terms of polysaccharide extraction yields (Table 1), *D. dichotoma* produces alginate at a rate of 14.15 ± 0.19%. However, this figure varies significantly depending on the collection location, with a notably higher yield of 18.73% observed on the Indian Gujarat coast [32], contrasting starkly with a lower yield of 8.8 ± 2.12% when collected on the Sudanese Red Sea coast [33]. In our current investigation, *S. muticum* yielded an alginate content of 17.40 ± 0.95%, quite high compared with *S. muticum* (10.23%) sampled in Spain [34].

Furthermore, our findings indicate that *C. officinalis* produces agar-agar at a rate of 8.85 ± 0.29%, contrasting with the significantly higher yield of 36.57 ± 1.06% from *C. officinalis* collected on the Egyptian Mediterranean coast [35].

In our study, *C. officinalis* yielded approximately 8.19 ± 0.18% carrageenan. This rate is relatively low compared to *E. elongata* (14.2%) collected during the low tide period in March 2015 on the Pamban and Manapdu coasts in India [36], as well as to the carrageenan extracted from *Corallina officinalis* (4.82 ± 1.52%) collected in a rocky shore basin near Skagaströnd (north-west Iceland) in October 2020 [37].

Ulvan production from *U. lactuca* was 2.47 ± 0.10% (Table 1). This result is slightly lower than other ulvan extraction yields in various studies, such as ulvan from *U. fasciata* (6.87 ± 1.21%) found in Abu Quir Bay, Egypt [38], and from *U. lactuca* (4.69 ± 0.76%) collected in Ho-Ping, Keelung, Taiwan [39].

In our current study, *C. laetevirens* had an ulvan extraction yield of 2.78 ± 0.07%, slightly lower than the ulvan extraction yield of *C. aerea* collected in Yantai, China, at 5.65% [40].

Based on these findings and comparisons, it is clear that the spatial and temporal variability in the bioactive compound’s composition of the collected seaweed is primarily caused by local environmental factors such as nutrient availability, suspended particles, and, thus, light availability, which affect substantially the chemical composition of seaweed biomass.

The water’s residence time may also impact the seaweed composition, which determines the likelihood that nutrients will be converted into new biomass [41]. This study demonstrated spatial synchronicity in seaweed fractions where seasonal differentiation was typically possible, independent of the sampling site. According to Breuer et al. [42], the bioactive compounds of the different seaweed groups (environmental and structural parameters) were most influenced by climate, nutrients, flow, and light regime. There is currently no consensus on the variables that control the composition of bioactive compounds in seaweed communities. Therefore, the additional information in this study is useful for understanding ecosystem functioning as well as the ecological assessment and modeling of seaweed. It is necessary to conduct additional research, particularly in other ecoregions, to confirm the results of this study and draw broader conclusions about the five seaweed communities under study. Future studies should concentrate on how environmental influences affect the seaweed communities being further investigated and understood. Local site-specific factors affect the composition of seaweed bioactive compounds, especially short-term temporal dynamics, despite being constrained by primary agents. Therefore, various scales of environmental and structural parameters should be included in investigations.

### 2.2. Seaweed Extracts Analyzed by FT-IR

Various phytochemical constituents in raw seaweed extracts can be confirmed by qualitatively analyzing multiple functional groups and extracts using Fourier transform infrared (FTIR) spectroscopy.

**Figure 1:**
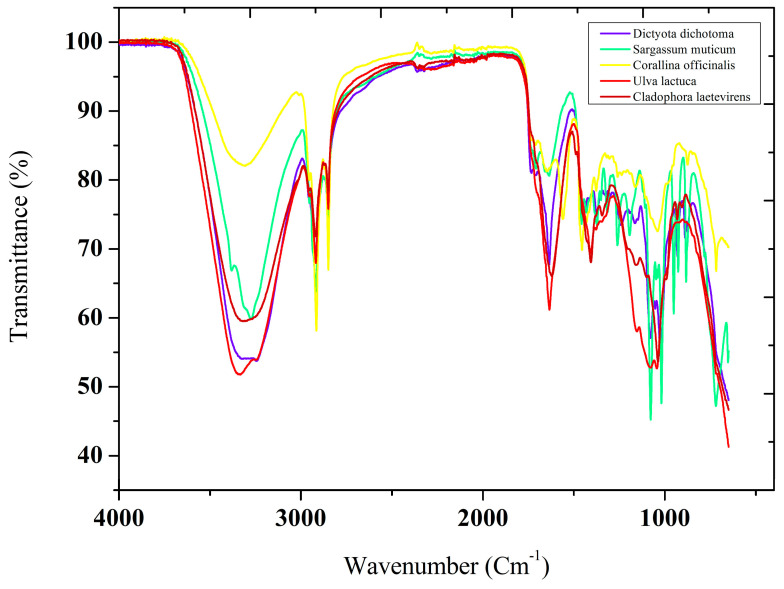
FT-IR spectra of the five studied seaweeds that were collected from the northeastern coast of Algeria.

As shown in Figure 1, FT-IR spectra of the five kelp species studied, collected on the northeast coast of Algeria, reveal the presence of several functional groups, including alkanes, aliphatic compounds, carbonyls, alcohols, and phenols. Carbon-carbon double bonds (C=C), alkenes and carbon-chlorine bonds (C-Cl), and other functional groups are among those identified. The spectra’s allure is nearly identical, in the range from 3600 to 3200 cm^−1^, and the vibrational frequencies of the hydrogen-bonded O-H stretch were observed in the functional groups of alcohols and phenols [43]. C-H stretch vibration frequencies were present in the functional groups of alkanes and aliphatic components. Values varied slightly between species but ranged from 2906 to 2922 cm^−1^ [44]. Carbonyls were observed in the 1709 to 1733 cm^−1^ range. C=C double bonds were characteristic of aromatic carbonyls, with vibrational frequencies between 1620 and 1652 cm^−1^ [45]. Alkenes showed C-H bending vibration frequencies between 880 and 952 cm^−1^, while C-Cl bonds were observed at 716 cm^−1^ in *S. muticum* and 727 cm^−1^ in *C. officinalis* [46].

### 2.3. Seaweed’s Elementary Analysis Using X-ray

By performing a fundamental analysis, X-ray fluorescence spectroscopy is a vital tool that enables us to delve deeper into the variations between seaweed species and their constituent elements. The values for individual elements that have the same letter indices (e.g., “a”) indicate that there are no statistically significant differences between them. However, subsequent letter indices (a, b, c, d, e) determine groups in descending order. According to the study’s findings, the nutritional makeup of the five seaweed species under investigation differs significantly. For their use in various fields, these variations in nutritional profiles have significant ramifications. In particular, potassium (K), magnesium (Mg), sulfur (S), and calcium (Ca) are crucial for the development of algae (Table 2). For the content of Rb, Cu, As, and Mn, no significant differences between seaweed species were observed (Table 2).

The highest magnesium (Mg) level is found in *D. dichotoma* (3.96 ± 0.34%), followed by *S. muticum* (2.20 ± 0.386%). These results align with those attained for *D. dichotoma* (17.17 mg.g^−1^) and *Sargassum odontocarpum* (formerly *S. coriifolium*) (15.45 mg·g^−1^) collected on the Caribbean Sea island of St. Martin [47]. Magnesium is necessary for the production of chlorophyll and other metabolic processes, which may be advantageous for use in agriculture and nutrition [48]. *Dictyota dichotoma* (4.93 ± 0.013%) has a significantly higher sulfur (S) content than *S. muticum* (2.82 ± 0.011%). However, the latter contains more sulfur than the *Sargassum* (0.82 ± 0.22%) collected in Barbados [49]. Additionally, *D. dichotoma* has a silicon (Si) level that is much lower (0.959 ± 0.017%) than that of *S. muticum* (4.100 ± 031%). Silicon’s potential role in cell structure and resistance to environmental stress makes it potentially useful for agricultural and pharmaceutical applications [50].

When compared to *C. officinalis* (0.861%) collected in Holbeck, North Yorkshire, UK, the calcium (Ca) content of the same species in this study is relatively high, reaching 36.48 ± 0.035% [51]. This seaweed also includes aluminum (Al) (2.39 ± 0.056%) and silicon (Si) (3.06 ± 0.031%), but neither phosphorus nor zinc are found. It also has low contents of potassium (K) (0.455 ± 0.004%), iron (Fe) (0.379 ± 0.009%), sulfur (S) (1.03 ± 0.008%), and manganese (Mn) (0.015 ± 0.003%).

The results of an elemental analysis of green seaweed show that the elements in those organisms differ significantly. Many metabolic processes, including seaweed growth, depend heavily on potassium. *Ulva lactuca* (3.32 ± 0.010%) and *C. laetevirens* (2.31 ± 0.007%) have equivalent concentrations of potassium (K). In addition, *C. laetevirens* has significantly higher magnesium (Mg) (6.26 ± 0.269%) and sulfur (S) (13.65 ± 0.022%) contents than those of *U. lactuca* (1.61 ± 0.361% and 6.62 ± 0.016%, respectively, for Mg and S).

The composition of *C. laetevirens* is quite similar to that of *C. glomerata* collected in Iran [52]. Calcium and potassium were also detected in *U. lactuca* harvested on the island of Qheshm (in the Persian Gulf) in southern Iran [53]. Calcium is essential for dental and bone health, as well as for various physiological processes. The variations in the fundamental composition of the multiple seaweeds provide numerous options for different applications. Each type of seaweed has distinct uses depending on the specific elements present, so to fully exploit their potential, it is essential to understand their composition. Many industries, including human food, agriculture, medical testing, and other areas of interest, can see these advantages.

### 2.4. Seaweed-Based Analysis by UPLC-ESI-MS/MS

Brown seaweeds, such as *D. dichotoma* and *S. muticum*, were very rich in polyphenols and vitamins, as shown for the methanolic extract, with chrysin accounting for the majority, at 52.52% and 53.60% for *D. dichotoma* and *S. muticum*, respectively. Chrysin has also been detected in *D. cervicorni* in Saudi Arabia’s Red Sea [54]. Vanillin levels are similar for both algae, varying between 13 and 14%. Vanillin is a compound quantified in *Sargassum wightii* harvested in India [55].

A low flavonoid concentration was noticed: kaempferol, which accounts for 3.55% of D. *dichotoma* and esculin for 4.26% of *S. muticum*. If not identical, the ethyl acetate and n-butanol fractions have remarkably similar compositions. Flavonoids are available in trace form, with the compound with the highest content being kaempferol for all fractions of *D. dichotoma* and Hespertin for the ethyl acetate and n-butanol fractions of *S. muticum*, the latter also making up a variety of Moroccan brown seaweed [56].

The red seaweed *C. officinalis* is famous for having gallic and chlorogenic acids in it. However, this study found no gallic acid, and the ethyl acetate and n-butanol fractions only contained trace amounts of chlorogenic acid. Additionally, as polyphenols, the methanolic fraction includes ascorbic acid (17.92%) and vanillin. (13.72%). Flavonoids and esculin are only present in trace amounts (3.59%). In both fractions, benzoic acids were present at 47–56%.

**Figure 2:**
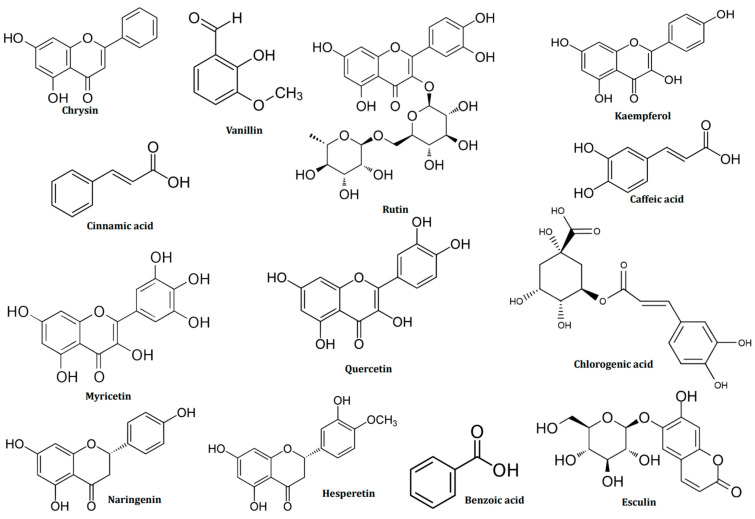
Chemical structure of identified seaweed bioactive compounds.

There are differences in the composition of the green seaweed, *U. lactuca*, which was the subject of this study. Hespertin, which makes up 3.93% of the methanolic fraction of *C. laetevirens*, is a polyphenol. The last 9% comprises naringenin, quercetin, rutin, cinnamic acid, caffeic acid, and 4-hydroxy-coumaric acid. This bioactive composition is quite similar to that obtained from *C. glomerata*, where rutin, quercetin, and kaempferol harvested in Thailand could be detected [57]. On the other hand, the methanolic fraction of *U. lactuca* corresponds better to that of the brown and red seaweed studied. Vanillin makes up 39.32% of its composition, making it a predominant polyphenol. The results of Kumar et al. for *Ulva rigida* [55] and esculin as a coumarin at 9.51% are equivalent to those of this study. The amounts of benzoic acid and hesperine in the ethyl acetate and n-butanol fractions of the two seaweeds are still comparable, ranging from benzoic acid (16–19%) to hespertin (3–4%). The results are consistent with those found in samples of *U. fasciata* collected in the Mediterranean [58] and *C. glomerata* collected in Poland [59]. The following figure (Figure 2) displays the chemical structures of the major bioactive compounds identified in the methanolic extracts of the brown algae *D. dichotoma* and *S. muticum*, the red algae *C. officinalis*, the green algae *U. lactuca*, and *C. laetevirens*. The overall data for the analysis are provided in the Supplementary Materials in Tables S1–S5 and Figures S1–S5. These findings suggest that, with a few minor exceptions, the composition of the studied seaweed is mainly comparable. This suggests that their growth environment, including their shared geographic location, environmental conditions, and interactions with other organisms, impacts them [60,61].

### 2.5. Assessment of the Nutritional Compounds

The results of the primary composition of the raw powdered five-seaweed sampled from the northeastern Algerian coast are summarized in Table 3. The highest content of lipids and total sugars was obtained in the extract from *Dictyota dichotoma*, the highest soluble protein was in *Cladophora laetevirens*, and the lowest concentration occurred, respectively in Ulva lactuca and *Corallina officinalis.* Proteins were lowest in *Sargassum muticum*, while total sugars were lowest in *Ulva lactuca*.

#### 2.5.1. Lipid Content

The lipid content of seaweeds can vary significantly between species within the same genus, influenced by their respective environments. Our findings reveal notable disparities in the lipid content among the five seaweed samples examined. Among brown seaweed, *D. dichotoma* shows the highest lipid percentage, with an average of 3.07% ± 0.13 (dry weight). This aligns with a previous study [62], which reported a lipid content of 3.74% ± 0.01 from the same species sampled in the summer on the İskenderun Bay coast in Turkey. These species therefore present an interesting potential in terms of lipid yield and have potential applications in various fields, including biofuel production and functional food ingredients. In contrast, *S. muticum* shows a much lower lipid percentage, with only 0.34% ± 0.06. Although this percentage is significantly lower than that of *D. dichotoma*, it remains pertinent, especially concerning its applications in human and animal food. *Coralina officinalis* and *U. lactuca* show even lower lipid percentages, both at 0.16% ± 0.01 and 0.16% ± 0.02, respectively. Considering the green seaweed, *C. laetevirens*, it shows an intermediate percentage of 0.5% ± 0.03, which is considered a moderate lipid percentage and potentially useful in various fields, but further research is needed to determine its optimal use. The fluctuations in the lipid composition of seaweed underscore the influence of specific environmental factors, including light, nutrients, pollution, and salinity [63,64]. With regard to winter harvesting, it is important to note that algae can accumulate lipids in greater quantities during cold months, as has been observed in *D. dichotoma*. This lipid accumulation may be an adaptive response to more stressful environmental conditions, such as lower temperatures. This observation underlines the importance of considering specific environmental conditions when interpreting algal lipid content data.

#### 2.5.2. Protein Content

Seaweed protein constitutes a vital component of its nutritional profile, offering crucial insights into its potential as a food and nutritional resource. Our findings unveil a broad spectrum of protein content across all analyzed seaweed species. *Cladophora laetevirens* has the highest percentage of soluble protein, with an average of 5.15% ± 0.002 (dry weight). This species therefore stands out for its high protein content, making it an interesting candidate for various applications, notably in human food, aquaculture, or even medical and pharmaceutical fields. In comparison, *C. officinalis* also shows a high soluble protein content, with an average of 4.71% ± 0.004. *Cladophora laetevirens*. *D. dichotoma*, *S. muticum*, and *U. lactuca* show slightly lower soluble protein percentages than the first species, although they are relatively similar to each other. *Dictyota dichotoma* shows 4.51% ± 0.005, *S. muticum* shows 4.32% ± 0.003, and *U. lactuca* shows 4.49% ± 0.002. These results underscore the significant variability in seaweed protein content and emphasize the influence of environmental conditions on their nutritional composition. Seaweed collected at greater depths typically exhibits higher protein content than those collected at the surface, attributable to reduced light exposure, increased nutrient availability, and environmental factors unique to deep-sea habitats [65,66].

#### 2.5.3. Total Sugar Content

Evaluating the total sugar content of seaweed provides crucial insights into its nutritional profile and potential as a carbohydrate source. Our study underscores significant variations in the total sugar content among the five seaweed species analyzed. *Dictyota dichotoma* has the highest percentage of total sugars, with an average of 57.87% ± 0.04 (dry weight). This species therefore stands out for its high sugar content, making it an interesting potential source for the production of bioethanol, biomaterials, or even food ingredients. In comparison, *S. muticum* also displays a significant total sugar content, with an average of 46.43% ± 0.12. Although slightly lower than that of *D. dichotoma*, this value remains significant and suggests that *S. muticum* could also be exploited in various applications. *Cladophora laetevirens* and *C. officinalis* show intermediate percentages of total sugars, with 40.67% ± 0.09 and 34.93% ± 0.11, respectively. Although these values are lower than those of *D. dichotoma* and *S. muticum*, they are still applicable for the same applications. On the other hand, *U. lactuca* had the lowest percentage of total sugars among the species studied, with an average of 24.53% ± 0.04. This species may be less interesting for large-scale sugar production but could nevertheless have other applications, such as biofertilizer production or bioremediation. These findings illustrate significant variations in seaweed total sugar content, indicating their diverse composition and potential as food and nutritional carbohydrate sources.

### 2.6. Assessment of the Total Phenolic and Total Flavonoid Compounds

Secondary metabolites, such as phenolic and flavonoid molecules, play indirect roles in physiological processes [67]. The collective concentration of total phenolic and flavonoid compounds in the diverse crude and fractionated extracts from the five seaweeds investigated in this study is detailed in Table 4. The total content of phenolic compounds, expressed in µg GAE·mg^−1^, as well as the content of flavonoids, in µg QE·mg^−1^, differed significantly between algae species.

#### 2.6.1. Total Phenolic Content

The results presented in Table 4 displayed that the highest contents of polyphenols were obtained using EtOAc extracts from all five seaweeds studied here. Seaweed can be classified according to their phenolic content in the following decreasing order: *S. muticum* (235.67 ± 1.13 µg GAE·mg^−1^), *C. laetevirens* (215 ± 2.33 µg GAE·mg^−1^), *C. officinalis* (211.04 ± 2.35 µg GAE·mg^−1^), *D. dichotoma* (189.33 ± 3.11 µg GAE·mg^−1^), and *U. lactuca* (158.89 ± 2.79 µg GAE·mg^−1^). *S. muticum* stands out with the highest phenolic compound content, reaching 235.67 ± 1.13 µg GAE·mg^−1^. These results are comparable to those of *Sargassum* sp. (212.8 mg EAG·g^−1^) collected in Indonesia [68] but significantly lower than the ethyl acetate of *S. muticum*, which was collected from Morocco (21.63 ± 0.270 mg EAG·g^−1^) [69]. *Corallina officinalis* and *C. laetevirens* also show significant concentrations of phenolic compounds, with values of 211.04 ± 2.35 µg GAE·mg^−1^ and 215 ± 2.33 µg GAE·mg^−1^, respectively. These results suggest that these seaweed species could be interesting candidates for pharmaceutical or cosmetic applications because they are a source of phenolic compounds with high bioactive potential, given their potentially beneficial health properties. Conversely, *U. lactuca* showed the lowest content among the samples tested, with 158.89 ± 2.79 µg GAE·mg^−1^. However, this value remains significant and could be exploited in several applications. It is important to note that these variations in phenolic compound content can be influenced by several factors, including seaweed species, environment, and harvesting and extraction conditions.

#### 2.6.2. Total Flavonoid Content

Data on flavonoid levels in seaweed extracts reveal significant variations between the species studied. The highest flavonoid concentrations were extracted with EtOAc from the five seaweeds studied (Table 4). The seaweed *C. laetevirens* has the highest flavonoid content, with an impressive concentration of 331.05 ± 3.11 µg EQ·mg^−1^. This species clearly stands out from the others in terms of flavonoid content and could be an important source of these beneficial compounds. This result surpasses that of *C. aegagropila* obtained from the Black Sea with 39.5 ± 0.4 mg EQ·g^−1^ [70] and *C. glomerata* studied in India, which has a total flavonoid content of 70.49 ± 2.91 mg EQ·g^−1^ [71]. *Sargassum muticum* also has a relatively high flavonoid content, with 175.01 ± 0.87 µg EQ·mg^−1^. This seaweed could therefore also be promising for the isolation of flavonoids for pharmaceutical or cosmetic purposes. *Ulva lactuca* had an intermediate flavonoid content among the samples tested, with 112.05 ± 1.89 µg EQ·mg^−1^. Although this value is lower than that of *C. laetevirens* and *S. muticum*, it remains significant and deserves to be considered when exploring the biotechnological applications of seaweed. Conversely, *C. officinalis* had the lowest flavonoid content of all the samples tested, with just 75.41 ± 1.87 µg EQ·mg^−1^. This species may require further attention to determine whether it contains other bioactive compounds of interest. *Dictyota dichotoma* shows a moderate flavonoid content, with 98.45 ± 1.12 µg EQ·mg^−1^. Although this value is lower than that of some other species, it remains significant. The results underline the potential of seaweed as a source of flavonoids, valuable compounds for various biotechnological applications. *Cladophora laetevirens* stands out with an exceptionally high flavonoid content, offering an interesting opportunity for their large-scale extraction.

### 2.7. Antioxidant Activity

The antioxidative effects of the seaweed extracts are anticipated to be investigated utilizing DPPH, ABTS, reducing power, silver nanoparticles, and phenanthroline tests due to the complexity of the phytochemical components. Table 5 presents the findings of these tests in a more comprehensive manner that is simpler to recall, comprehend, and correlate with other substances.

#### 2.7.1. ABTS Radical-Scavenging Activity

Like the DPPH test, the ABTS^+^ test evaluates the capacity of hydrogenated antioxidants to bind the ABTS radical in solution and inhibit lipid oxidation via chain-break antioxidants. *Cladophora laetevirens* 78.65 ± 0.96, *Ulva lactuca* 102.74 ± 1.89, *Corallina officinalis* 127.05 ± 1.89, *Dictyota Dichotoma* 129.28 ± 1.78, and *Sargassum muticum* 389.11 ± 2.13 had the highest ABTS^+^ scavenging capacity among the various seaweed extracts. The highest concentration was attributed to *C. laetevirens*, specifically the EtOAc extract (IC_50_ = 78.65 ± 0.96 µg·mL^−1^). The results obtained in this study for the green seaweed are superior to those obtained for *Cladophora glomerata* in Thailand (IC_50_ = 65.21 ± 0.55 µg·mL^−1^) [57]. The seaweed *D. dichotoma* (IC_50_ = 162.89 ± 2.46 µg·mL^−1^) is somewhat higher than that studied by El-Shaibany in Yemen (IC_50_ = 204.60 ± 8.30 µg·mL^−1^) [72]. In contrast to the brown seaweed *S. muticum*, the seaweed *S. vulgare* (IC50 = 72.9 ± 5.83 µg·mL^−1^) collected from the same location [73] exhibits a stronger anti-radical capacity than the other algae investigated in the current study.

#### 2.7.2. DPPH Radical-Scavenging Activity

The neutralizing abilities of antioxidants are frequently evaluated using the stable free radical DPPH. The ability of seaweed extracts to act as hydrogen donors and antioxidants is assessed by this test. Table 5 displays the levels of DPPH activity in various extracts for the seaweed under investigation. The green seaweed *C. laetevirens*, which had the highest rate for this study at 89.11 ± 0.98 g·mL^−1^ for EtOAc, was the first to exhibit any significant DPPH activity. The brown seaweed *D. dichotoma* and *S. muticum* achieved IC_50_ values of (288.56 ± 2.98 µg·mL^−1^) and (276.23 ± 1.98 µg·mL^−1^), respectively. These inhibition concentrations are better than those obtained for *D. dichotoma* (IC_50_ = 458.24 ± 2.98 µg·mL^−1^), found in the Gulf of Mannar, between southeast India and eastern Sri Lanka [74]. The highest effective concentration ever observed was even found in the study by Prasedya et al. [75] for several species of *Sargassum* sp. (IC_50_ = 737.30 ± 23.46 g·mL^−1^).

#### 2.7.3. Reducing Power Activity

Using the reduction method, the antioxidant capacity of seaweed was evaluated. *S. vulgare* and *D. dichotoma* collected from the Mediterranean Sea in Algeria did not exhibit antioxidant activity at a dose of 4 mg·mL^−1^ [73]. However, compared to other seaweeds like *U. lactuca* and *C. laetevirens*, they showed a lower reduction capacity than that of other seaweeds such as *C. glomerata* (A_0.5_ = 71.75 ± 0.14 µg·mL^−1^) in Thailand [57].

#### 2.7.4. O-Phenanthroline Chelating Activity

O-Phenanthroline activity is a crucial technique for assessing antioxidant activity. In this process, 1,10-o-phenanthroline and Fe^2+^ react rapidly to create a highly stable red complex. According to a summary of the concentrations in Table 5, the effective doses for which absorbance was 0.5 were as follows: *U. lactuca* 5.67 ± 0.82 µg·mL^−1^, *C. laetevirens* 10.06 ± 0.88 µg·mL^−1^, *S. muticum* 72.07 ± 1.89 µg·mL^−1^, *C. officinalis* 83.45 ± 0.88 µg·mL^−1^, and *D. dichotoma* 85.71 ± 1.32 µg·mL^−1^. The O-phenanthroline activity in this study demonstrated that ethyl acetate extracts from green seaweed had a significant capacity to absorb ferric ions. *S. vulgare*, harvested in the same area as our research, had a lower concentration (A_0.5_ (µg·mL^−1^) > 200) [73], but *S. muticum* had a higher concentration (A_0.5_ = 170.28 ± 0.96 µg·mL^−1^).

#### 2.7.5. Silver Nanoparticles

Silver nanoparticles (SNPs) are formed from silver salts by reducing Ag^+^ ions to Ag^0^ silver nanoparticles. All five algae studied showed this ability. Ethyl acetate extracts showed the highest concentrations, followed by n-butanol and crude methanol extracts. Antioxidant activity varied from *C. officinalis* 11.58 ± 0.79 µg·mL^−1^, *C. laetevirens* 24.57 ± 1.03 µg·mL^−1^, *S. muticum* 45.79 ± 1.21 µg·mL^−1^, *U. lactuca* 98.32 ± 1.87 µg·mL^−1^ and *D. dichotoma* 109.87 ± 1.89 µg·mL^−1^.

### 2.8. Correlation between Antioxidant Assays

In this study, we examined the antioxidant activity, total flavonoid content (TFC), and total phenolic content (TPC) of the five seaweeds.

The mean values of antioxidant activity for the DPPH, ABTS, and reducing power tests are 413.01, 203.65, and 198.46, respectively. The median for each of these tests indicates variation in antioxidant activity among the samples.

The standard deviation for each test shows that the data deviate from the mean, suggesting significant variability in antioxidant activity among the tested samples. Kurtosis and skewness suggest the shape of the data distribution, with higher values for the ABTS and phenanthroline tests potentially indicating a more concentrated but asymmetric distribution.

The range of values for each test is significant, indicating large variability in antioxidant activity among the samples. The coefficients of variation are high, confirming substantial variability in the data for each of the tests. The descriptive statistics presented in the supplementary file (Tables S6 and S7) provide a detailed analysis of total phenolic and flavonoid content, as well as the various antioxidant activity tests. Table 6 presents the results regarding the antioxidant activity, total flavonoid content (TFC), and total phenolic content (TPC) in various samples.

Table 7 shows Pearson’s simple correlation coefficients between different antioxidant assays, including DPPH, ABTS, reducing power, phenanthroline, and SNP.

The DPPH and ABTS show a slight positive correlation (0.18), suggesting that there is some, although limited, similarity in the results of the two tests.

Power reduction has a moderate positive correlation with DPPH (0.36) and a small positive correlation with ABTS (0.30), indicating some commonalities between these tests.

Phenanthroline test results have a small positive correlation with both DPPH (0.23) and ABTS (0.60), suggesting that these tests measure different aspects of antioxidant activity.

The SNP shows a slight positive correlation with DPPH (0.39) and potency reduction (0.38), suggesting some similarity in the results of these assays but little association with other antioxidant assays. Overall, the table suggests that different antioxidant tests may have some common characteristics but also measure different aspects of antioxidant activity.

Table 8 presents Pearson’s simple correlation coefficients, illustrating the relationships between antioxidant activity and bioactive compounds.

DPPH, being compared to itself, naturally shows a perfect positive correlation (1.00).

There is a negligible negative correlation (−0.05) observed between DPPH and ABTS, suggesting a slight inverse relationship between these two antioxidant assays.

A modest positive correlation (0.14) is found between DPPH and reducing power, indicating a tendency for samples with higher DPPH activity to also exhibit higher reducing power.

Phenanthroline displays a weak positive correlation with DPPH (0.02), a moderate positive correlation with ABTS (0.52), and a moderate positive correlation with reducing power (0.45). This suggests that samples with higher levels of phenanthroline tend to have higher antioxidant activity in these assays.

SNP demonstrates a weak positive correlation with DPPH (0.22) but a weak negative correlation with ABTS (−0.12), suggesting a slightly different relationship between SNP and the two antioxidant assays.

Total flavonoid content (TFC) exhibits a strong negative correlation with DPPH (−0.70), implying that samples with higher levels of flavonoids tend to have lower DPPH activity, indicating a potentially inhibitory effect of flavonoids on DPPH radical scavenging.

Total phenolic content (TPC) shows a moderate negative correlation with DPPH (−0.43) and a moderate positive correlation with phenanthroline (0.57), suggesting some degree of association between TPC and these antioxidant assays.

In summary, the correlations between antioxidant activity and bioactive compounds vary in strength and direction, indicating complex relationships between these variables.

### 2.9. Antibacterial Activity

The study revealed an increase in antimicrobial activity as the concentration of extract increased, indicating a possible correlation between concentration and effectiveness. Antimicrobial activity results for the different species are summarized in Table 9.

The results are also significantly influenced by the type of bacteria used in the test. When tested against *E. coli*, *D. dichotoma* displayed marginally higher antibacterial activity than other algae at doses of 1000 g·mL^−1^ and 500 g·mL^−1^. Although, to a lesser extent, *D. dichotoma* also demonstrated above-average performance against *S. aureus*. These results are consistent with those of the same species studied by Imran et al. [46]. The performance of *S. muticum* consistently remained stable when tested against E. coli at all concentrations. However, it appears less effective against *S. aureus* than *D. dichotoma*, particularly at lower concentrations. The results are superior to those found for *S. fusiforme* and *S. oligocystum* collected along the Zhejiang coast in China [76] and Cagayan, Philippines [77], respectively.

As the concentration diminished, *C. officinalis*’ antimicrobial activity toward *E. coli* decreased. Its performance against *S. aureus* was comparable to that of *S. muticum*, albeit somewhat lower at lower concentrations. These results are similar to those of *C. elongata* collected in Mostaganem, Algeria [78]. *U. lactuca’s* antimicrobial properties towards *E. coli* are moderate but diminish with decreasing concentrations. When tested against *S. aureus*, it appears slightly less potent than *D. dichotoma* at all concentrations. *U. lactuca* collected in Algeria showed perfect activity (23.2 ± 0.46 mm) against *E. coli* and (13.8 ± 0.23 mm) against *S. aureus* [78]. In lower concentrations, *C. laetevirens*’ antimicrobial activity toward *E. coli* diminishes. As opposed to the other types of seaweed, it appears to be less effective against *S. aureus* when tested, especially at lower concentrations. However, *C. glomerata* collected in Iran showed weaker antimicrobial activity than *S. aureus* [79].

## 3. Materials and Methods

### 3.1. Sampled Seaweed

**Figure 3:**
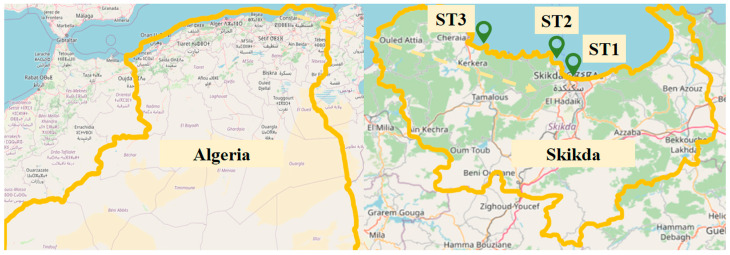
Overview of sampling locations.

Five species of seaweeds were collected from the surface of rocky substrates in the waters off the Skikda coast in northeastern Algeria to assess their algal abundance. The main morphological characteristics of the algae collected on Algeria’s northeast coast are detailed in Table 10. *Dictyota spiralis* (formerly *Dictyota dichotoma var. elongata*) (Kützing) Grunow and *Sargassum muticum* (Yendo) Fensholt were collected at the Stora site, Site 1 (latitude: 36°53′54.9″ N, longitude: 6°52′48.1″ E) in January 2022. *Corallina officinalis* Linnaeus was collected in March 2022 at the Ravin des Lions beach, Site 2 (latitude: 36.91185° or 36°54′43″ N, longitude: 6.88418° or 6°53′3″ E). *Ulva lactuca* Linnaeus and *Cladophora laetevirens* (Dillwyn) Kützing were collected in May and June, respectively, at Ain Lakssabe in the commune of Collo, Site 3 (latitude: 36.99044° or 36°59′26″ N, longitude: 6.56438° or 6°33′52″ E) (Figure 3). Seaweed species have been identified using taxonomic keys of seaweed commonly found in Mediterranean waters based on their physical traits [80,81,82]—seawater removed epiphytes, animal casts, sand, and other debris from the collected seaweed samples. The fresh biomass was thoroughly rinsed in fresh water to remove salt residue. To stop photolysis and thermal deterioration, the cleaned seaweed biomass was dried by air in the shade. The dry material was weighed and coarsely ground using a mechanical grinder before being kept in the dark in hermetically sealed bottles, free from moisture.

### 3.2. Extraction of Bioactive Compounds

The obtained seaweed powders were macerated in a hydro-methanolic solution (1:10) (powder (g)/solution (mL)) (4:1) (methanol/water) (*v*/*v*) at room temperature for 24 h under continuous agitation. This extraction process was replicated three times. To obtain the maximum extraction of bioactive compounds from the seaweeds, the solvent was refreshed with every essay to ensure consistent extraction conditions. The resulting suspensions were filtered through 22 µm pore size filters (Whatman^®^ ashless filters, Grade 541, Whatman, London, UK). The filtrate was evaporated at 40 °C using a rotary evaporator (Laborota 4000, Heidolph Instruments GmbH & Co. KG, Schwabach, Germany). After evaporating the filtrate, the resulting residue was dissolved in water and subjected to a multi-step extraction process using solvents of increasing polarity. A portion of the resulting extract was then diluted in 100 mL of distilled water and subjected to a liquid-liquid extraction. First, the dissolved residue underwent extraction with hexane, followed by ethyl acetate, and finally, n-butanol. Each extraction was performed to ensure comprehensive extraction of the different compounds present in the seaweed powder. This step further refined the extract, allowing for the separation of compounds based on their solubility in the respective solvents. Solvents were evaporated under pressure in a rotary evaporator set to 50 °C. Two fractions were obtained after evaporation: ethyl acetate EtOAc and n-butanol n-BuOH.

### 3.3. Polysaccharide Extraction

#### 3.3.1. Extraction of Alginate

Alginates were extracted from the seaweeds *D. dichotoma* and *S. muticum* following the method of Torres et al. [83], with some adjustments. The seaweed was treated by soaking in a 2% formaldehyde solution for 24 h, followed by repeated washing with distilled water and filtration. The residue was incubated in 0.02 N sulfuric acid at 60 °C for 2 h with constant stirring, followed by thorough washing with distilled water and filtration. The recovered particles were then carbonated in a 4% sodium carbonate solution at 60 °C for 2 h, followed by filtration. The filtrate containing the polysaccharides was dialyzed for 48 h using a dialysis membrane with a cut-off of 3500 Da. The polysaccharides were thus purified before being dried. Finally, the sample was dried to obtain alginate.

#### 3.3.2. Extraction of Agar

Agar was extracted from *Corallina officinalis* according to the method mentioned by Marinho-Soriano [84], which involved heating pre-ground seaweed in distilled water to pH 6.5 at 130 °C under mechanical agitation. The extract was filtered and then gelatinized at room temperature for 12 h, followed by a freezing and thawing step. The resulting gel was washed to remove impurities, dialyzed (molecular weight cut-off: 3500 Da), and then dried at 60 °C for 24 h to obtain agar.

#### 3.3.3. Extraction of Carrageenan

Carrageenan was extracted from the red seaweed *C. officinalis*, according to the method of Gonzaler-Lopez et al. [85]. The seaweed was heated in an autoclave at 130 °C for 2 h, then cooled to room temperature. Vacuum filtration yielded a liquid phase containing liquid carrageenan, obtained through precipitation by adding 99% ethanol in a ratio of 1:1.5. No alkaline treatment was used to preserve the natural rheological properties of the extracted carrageenan. The precipitates were separated by vacuum filtration; the solid residues were washed twice with ethanol, and then the samples were dried at 40 °C for 24 h.

#### 3.3.4. Extraction of Ulvan

Ulvan was extracted from the green seaweeds *U. lactuca* and *C. laetevirens* using the protocol of Robic [86] with a few modifications. The seaweed was submerged in a sodium oxalate (1 L) solution at 130 °C for 3 h in an autoclave. The filtrate was dialyzed in distilled water for 48 h using Spectra/Por tubes and then precipitated in 99% ethanol. After one night at room temperature, the precipitate was recovered, washed twice in absolute ethanol, and dried at 50 °C.

### 3.4. Elemental Composition of Raw Seaweed

A Fourier transform infrared spectroscopy (FT-IR) analysis in the range 4000–600 cm^−1^ was used to determine the components of raw algae extracts in transmission mode. The spectra were gathered using a Thermo Scientific Nicolet iS50 (USA) FTIR spectrometer (Madison, WI, USA).

Raw seaweed powder was pelletized before being placed in the X-ray fluorescence spectrometer (SciAps, Woburn, MA, USA), which was used to determine the elemental composition.

The phytochemical analysis was performed by Ultra-Performance Liquid Chromatography-Electrospray Ionization-Tandem Mass Spectrometry (UPLC-ESI-MS/MS, Kyoto, Japan). Using a UPLC-ESI-MS-MS Shimadzu 8040 UltraHigh (Kyoto, Japan) sensitivity with UFMS technology equipped through a binary bomb Nexera XR LC-20AD, the quantification of various phytochemical compounds in crude algae extracts as well as the fractions n-BuOH and EtOAc of the examined seaweed was carried out. The separation was accomplished using an Ultra-force C18 column (I.D., 150 mm, 4.6 mm, 3 m particle size; Restek). The chromatographic separation was carried out using water and formic acid at 0.1% grade LC-MS as phase A and methanol grade LC-MS as phase B. The following gradient elution program was used: 80% A (0.1 min to 1 min), 20% A (1 min to 30 min), 0% A (30 min to 40 min), 0% A (40 min to 45 min), and 80% A (45 min to 60 min). The sampling rate was 0.03 mL·min^−1^, the injection volume was 5 µL through a Millex-LCR (PTFE) filter with a 0.22 mm pore size, and the column temperature was set at 30 °C. The ESI conditions used in the LC-MS-MS are as follows: 230 KPs of CID gas; −6.00 Kv conversion dynode; 350 °C interface temperature; 250 °C temperature DL; 3.00 L·min^−1^ gas flow; 400 °C thermal block; and 15.00 L/min gas flow. A mass spectrometer detected negative and positive ions in MRM mode (multiple reaction monitoring).

### 3.5. Determination of Polysaccharide, Carbohydrate, Total Phenolic, and Total Flavonoid Contents

#### 3.5.1. Yield Content

Yield is calculated by measuring the dry weight of algae before extraction and the weight of bioactive extracts and polysaccharides obtained after extraction. The following relationship gives the extraction rate or yield:

(1) $$Extraction\;yield\;\left(\%\right)=\frac{grams\;of\;extracted\;component}{grams\;of\;dry\;algae}*100$$

#### 3.5.2. Lipid Content

1 g of raw sample is placed in a cellulose cartridge of a “Soxhlet” extraction system; 25 mL of hexane is poured into aluminum crucibles; the extraction is completed by boiling the solvent and condensing its vapors using refrigeration; the extraction takes 55 min. The crucibles are then placed in an oven set to 105 °C for 24 h to entirely remove the remaining solvent [87]. The crucibles are then weighed again after cooling in a desiccator. The fat content is determined by:

(2) $$\left(\%\right)Lipid=\frac{\left(P_{f}-P_{i}\right)*100}{P_{0}}$$

***P*_0_**: Sample test point;
***P_i_***: Weight of the empty flask;
***P_f_***: Weight of the flask containing the fatty extract.

#### 3.5.3. Soluble Protein Content

The Bradford method was used to determine the soluble protein content of a standard range of Bovine Serum Albumin (BSA) [88]. A volume of 200 µL of dosing solution, 200 µL of Bradford’s reactant, and 1600 µL of ultra-pure water were added to glass test tubes. The mixture is homogenized in the vortex for 30 s. The absorbance is measured at 595 nm after 5 min.

#### 3.5.4. Sugar Content

The phenol/sulfuric acid method of Dubois et al. [89] was used for quantification to determine the total concentration in the crude extracts after 3 h at 105 °C with 1 N H_2_SO_4_ hydrolyzed. On the basis of a glucose standard, the results were calculated. After 5 min, the absorbance is measured at 490 nm.

#### 3.5.5. Total Phenolic Content

Utilizing the Folin–Ciocalteu reagent [90], the amount of total polyphenols is calculated using a dosage method on a microplate. A total of 20 µL of seaweed extract was combined with 100 µL of FCR diluted (1:10) and 75 µL of sodium carbonate (7.5%). This mixture was then left in the dark for 2 h at room temperature. By using a 96-well microplate reader, Perkin Elmer EnSpire (Singapore, Singapore), the absorbance was measured at 765 nm. In the same way, a blank is made by substituting the used solvent (methanol) for the extracted liquid. Using a calibration graph of gallic acid, the total phenol levels were defined as µg GAE.mg^−1^ extract.

#### 3.5.6. Total Flavonoid Content

The dosage of flavonoids in the extracts is based on the complex that forms between Al^+3^ and the flavonoids. With a few modifications, the Topçu [91] method was used to make a determination on a 96-well microplate. 50 µL of the extracted material is dosed and mixed with 130 µL of MeOH, 10 µL of potassium acetate (CH_3_COOK), and 10 µL of aluminum nitrate (Al(NO_3_)_2_, 9H_2_O) before being incubated for 40 min at room temperature. The absorbance at 415 nm was measured using a Perkin Elmer EnSpire (Singapore) device. Making a blank sample required mixing 50 µL of extrait with 150 µL of methane using the antioxidant quercetin as a standard. In accordance with the quercetin calibration curve, the data were expressed as µg quercetin equivalent per mg of extract (µg QE·mg^−1^).

### 3.6. Antioxidant Activity

#### 3.6.1. DPPH Radical-Scavenging Activity

Using the procedure described below and developed by Blois et al. [92], the DPPH free radical scavenging activity was assessed. The samples were mixed in various amounts with a 0.1 mM methanolic DPPH solution and left to sit at room temperature in the dark for 30 min. The results were represented as IC_50_ values, i.e., the concentration of the sample that can inhibit an enzyme by 50%, and were obtained using a microplate spectrophotometer (PerkinElmer, EnSpire, Singapore, Singapore).

#### 3.6.2. ABTS Radical-Scavenging Activity

The technique used in the ABTS decolorization experiment was developed by Re et al. [93]. The reaction between 2.45 mM potassium persulfate and 7 mM ABTS produced the cation ABTS^+^. After 24 h, the absorbance of the ABTS solution was modified to achieve an absorbance of 0.7000 ± 0.020 at 734 nm. Then, 40 µL of extracts at various concentrations were added to 160 µL of ABTS^+^ solution. The absorbance was determined at 734 nm, lasting 10 min in complete darkness. The results were presented as IC_50_ values.

#### 3.6.3. Reducing Power Activity

According to a study carried out by Oyaizu et al. [94], the reduced ability was dosed with potassium ferricyanide, and the absorbance was measured at 700 nm. Different extract concentrations were combined with tampon phosphate 0.2 M (pH 6.6) and 1% potassium ferricyanide K_3_Fe(CN)_6_ following a 20 min incubation at 50 °C. Then, 10 µL of ferric chloride FeCl_3_ (0.01%) and 10% trichloroacetic acid were added. The results were given as A_0.50_ μg.mL^−1^, the concentration that produced an absorbance of 0.5%.

#### 3.6.4. O-Phenanthroline Chelating Activity

The phenanthroline tests were carried out by Szydlowskaczerniak et al. [95]. For this test, 10 μL of extract at various concentrations was added to 50 μL of 0.2% ferric chloride FeCl_3_ solution and 30 μL of 0.5% phenanthroline solution. The reaction mixture was diluted to 200 μL with methanol, and absorbance at 510 nm was measured after 20 min at 30 °C. The findings were presented as absorbance values (A_0.5_ μg·mL^−1^).

#### 3.6.5. Silver Nanoparticles

To determine the metal chlorate activity, we followed the Mustafa Zyurek method described by Özyürek et al. [96]. It consists of converting silver ions (Ag^+^) into atomically small particles of silver (Ag^0^). Next, 20 µL of crude seaweed extract was added to 130 µL of SNP solution (50 mL) of AgNO_3_ (1.0 mM) and heated for 10 min. After that, 5 mL of trisodium citrate (1%) was added drop by drop until the color changed to pale yellow. Finally, 50 µL of H_2_O was added, and the mixture was incubated over 30 min at 25 °C. The read-out was performed at 423 nm. The results were presented as absorbance values (A_0.5_ μg·mL^−1^).

### 3.7. Antibacterial Activity

The antimicrobial activity of the five selected seaweeds was studied on two ATCC “American Type Culture Collection” bacterial strains, one Gram-positive *Staphylococcus aureus* ATCC^®^ 6538, and the second Gram-negative *Escherichia coli* ATCC^®^25922, both provided by the Algerian Pasteur Institute. An agar diffusion test was used to examine how bioactive seaweed extracts inhibited the growth of bacteria. Each bacterial strain was incubated in nutrient broth for 18 h at 37 °C. The bacterial inoculum solutions were made to have an optical density of between 0.08 and 0.1 D.O. at 620 nm. Transferred microorganism suspensions were evenly distributed across the Muller–Hinton agar surface of the plate. The seaweed extract solution was prepared using dimethyl sulfoxide (DMSO) with a purity of ≥99.9% (1000 μg·mL^−1^, 500 μg·mL^−1^, 250 μg·mL^−1^, and 125 μg·mL^−1^), and 30 µL of each solution was used to impregnate the disks with different concentrations of seaweed extract solution. Blank and serial dilution disks, along with positive and negative controls, were laid out on the agar surface. The negative control disks contained only the solvent, which is DMSO, while the positive control disks contained amoxicillin and ampicillin (30 µg per disc). Petri dishes were incubated at 37 °C for 24 h. After incubation, the inhibition zones formed around the disks were measured.

### 3.8. Statistical Calculations

The statistical analysis of the obtained test results was performed based on a one-factor or two-factor analysis of variance with repetitions (ANOVA) model and multiple Tukey’s least significant difference (LSD) tests [97]. The analyses focused on comparing the effects of algae species on the investigated variables. Tukey’s LSD tests allowed for detailed comparative analyses of means by identifying statistically homogeneous groups (homogeneous groups) and determining the so-called least significant differences (LSD). The ANOVA tables contain the most important elements of variance analyses, ending with the presentation of calculated probabilities (*p*-values) associated with the applied F-test functions (in the tables, *p* = Pr. > F) (Snedecor’s F or Fisher-Snedecor’s F). The calculated *p*-values determine the significance and magnitude of the effect of the examined factor on the differentiation of the analyzed variable results by comparing them with the most commonly accepted levels of significance (0.05). For detailed analyses based on Tukey’s multiple comparison tests, a significance level of *p* = 0.05 was adopted. The letter indicators next to the means determine the so-called homogeneous groups (statistically homogeneous). The occurrence of the same letter indicator next to the means (at least one) indicates no statistically significant difference between them. Subsequent letter indicators a, b, etc. specify groups of means in decreasing order. The LSD values serve as auxiliary measures, allowing for quantitative estimation of differences between means. Additionally, coefficients of variation (CV %) were calculated for each variable. They are measures of random variability in the conducted experiment [98].

## 4. Conclusions

The study examined the extraction efficiency of bioactive compounds, nutritional composition, and biological activities of five seaweed species collected from the northeast coast of Algeria. The results revealed significant variations between species in the previously mentioned terms. Among the extraction yields of bioactive compounds, *Dictyota dichotoma* stands out with the highest extraction yield of bioactive compounds (27.07%), followed by *Cladophora laetevirens* with a yield of 12.07%. In terms of polysaccharide yield, the two brown seaweeds stood out for their high alginate content, especially *Sargassum muticum* (17.40%). The seaweed studied showed outstanding nutritional composition, with varied lipid, protein, and carbohydrate profiles. *Dictyota dichotoma* had the highest percentage of lipids (3.07%), while *Cladophora laetevirens* had the highest soluble protein content (5.15%), and *Dictyota dichotoma* had the highest total sugar content (57.87%). These findings strongly show that the nutritional composition of seaweed varies depending on its environment. Chemical analysis using UPLC-ESI-MS-MS reveals the presence of various phenolic chemicals, flavonoids, and vitamins, highlighting the seaweed’s potential for use in food, medicine, and industries due to its diverse elemental composition. Finally, concerning biological activities, all the species studied showed the ability to scavenge ABTS^+^ and DPPH radicals, as well as reducing and chelating activity, especially *Cladophora laetevirens*, which showed excellent antioxidant activity (78.65 ± 0.96 µg.mL^−1^ for ABTS^+^ scavenging activity). The seaweeds *Dictyota dichotoma* and *Sargassum muticum* also demonstrated antibacterial properties, showing the highest performance against inhibition growth of *Escherichia coli* and *Staphylococcus aureus*. In conclusion, based on the obtained outcomes, seaweed from Algeria’s northeast coast offers significant potential as a source of bioactive compounds and beneficial nutrients for various applications, notably in the food, pharmaceutical, and cosmetics industries.

## Figures and Tables

**Table 1:** Bioactive compounds and polysaccharide extraction yields.

Seaweed	Yields (%)		
	Polysaccharide Extracts		Bioactive Extracts
*Dictyota dichotoma*	Alginate	14.15 ^b^ * ± 0.19 **	27.07 ^a^ ± 1.19
*Sargassum muticum*	Alginate	17.40 ^a^ ± 0.95	5.29 ^e^ ± 0.44
*Corallina officinalis*	Agar agar	8.85 ^c^ ± 0.29	6.11 ^d^ ± 0.12
	Carrageenan	8.19 ^d^ ± 0.18	
*Ulva lactuca*	Ulvan	2.47 ^d^ ± 0.10	9.55 ^c^ ± 0.12
*Cladophora laetevirens*	Ulvan	2.78 ^e^ ± 0.07	12.07 ^b^ ± 0.44
Mean		8.97	12.02
LSDp_0.05_		0.46	0.61

* The existence of identical letter indices in the averages (at least) indicates no statistically significant differences between them. Subsequent letter indices (a, b, c, d, e) define groups in descending order; ** Values are presented as means ± SD (n = 3).

**Table 2:** XRF determined the elementary analysis of the five sampled seaweeds. L.E.: light elements (carbon (C), hydrogen (H), nitrogen (N), oxygen (O)).

Element	*Dictyota dichotoma* (%)	*Sargassum muticum* (%)	*Corallina officinalis* (%)	*Ulva lactuca* (%)	*Cladophora laetevirens* (%)	Mean	LSDp_0.05_
**L.E.**	79.86 ^a^ * ± 0.000	76.26 ^a^ ± 0.000	52.64 ^e^ ± 0.000	74.76 ^b^ ± 0.000	70.00 ^d^ ± 0.000	70.70	3.68
**Aluminum (Al)**	1.20 ^d^ ± 0.035	2.08 ^b^ ± 0.045	2.39 ^a^ ± 0.056	1.71 ^c^ ± 0.038	0.945 ^e^ ± 0.032	1.67	0.09
**Phosphorus (P)**	0.184 ^b^ ± 0.005	0.155 ^c^ ± 0.006	Nd	0.230 ^a^ ± 0.006	0.086 ^d^ ± 0.005	0.13	0.01
**Potassium (K)**	6.58 ^b^ ± 0.012	7.23 ^a^ ± 0.013	0.455 ^e^ ± 0.004	2.31 ^d^ ± 0.007	3.32 ^c^ ± 0.010	3.979	0.207
**Iron (Fe)**	0.175 ^d^ ± 0.004	0.226 ^c^ ± 0.005	0.379 ^b^ ± 0.009	2.05 ^a^ ± 0.016	0.223 ^c^ ± 0.005	0.611	0.032
**Zinc (Zn)**	0.027 ^a^ ± 0.001	0.006 ^c^ ± 0.000	Nd	0.005 ^c^ ± 0.000	0.018 ^b^ ± 0.001	0.013	0.002
**Rubidium (Rb)**	0.006 ^a^ ± 0.000	0.012 ^a^ ± 0.000	0.005 ^a^ ± 0.000	0.009 ^a^ ± 0.000	0.004 ^a^ ± 0.000	0.007	ns ***
**Magnesium (Mg)**	3.96 ^c^ ± 0.340	2.20 ^d^ ± 0.386	4.31 ^b^ ± 0.433	1.61 ^e^ ± 0.361	6.26 ^a^ ± 0.269	3.67	0.19
**Silicon (Si)**	0.959 ^d^ ± 0.017	4.10 ^b^ ± 0.031	3.06 ^c^ ± 0.031	6.57 ^a^ ± 0.036	3.21 ^c^ ± 0.028	3.58	0.19
**Sulfur (S)**	4.93 ^c^ ± 0.013	2.82 ^d^ ± 0.011	1.03 ^e^ ± 0.008	6.62 ^b^ ± 0.016	13.65 ^a^ ± 0.022	5.81	0.30
**Calcium (Ca)**	2.02 ^d^ ± 0.006	4.69 ^b^ ± 0.010	36.48 ^a^ ± 0.035	3.67 ^c^ ± 0.008	2.21 ^d^ ± 0.007	9.81	0.51
**Copper (Cu)**	0.006 ^a^ ± 0.000	0.001 ^a^ ± 0.000	Nd	0.003 ^a^ ± 0.000	0.018 ^a^ ± 0.001	0.006	ns
**Arsenic (As)**	0.004 ^a^ ± 0.000	0.013 ^a^ ± 0.000	0.002 ^a^ ± 0.000	0.004 ^a^ ± 0.000	0.003 ^a^ ± 0.000	0.005	ns
**Strontium (Sr)**	0.090 ^c^ ± 0.000	0.160 ^b^ ± 0.001	0.179 ^a^ ± 0.001	Nd	0.007 ^d^ ± 0.000	0.087	0.005
**Titanium (Ti)**	Nd	0.031 ^d^ ± 0.008	0.050 ^c^ ± 0.013	0.338 ^a^ ± 0018	0.022 ^b^ ± 0.007	0.088	0.005
**Manganese (Mn)**	Nd	0.015 ^a^ ± 0.002	0.015 ^a^ ± 0.003	0.026 ^a^ ± 0.003	0.009 ^a^ ± 0.001	0.015	ns

* The existence of identical letter indices in the averages (at least) indicates no statistically significant differences between them. Subsequent letter indices (a, b, c, d, e) define groups in descending order; *** ns—not significant at p_0.05_. Nd: not detected.

**Table 3:** Primary composition of the five seaweeds collected from the northeastern Algerian coast.

Seaweed Species	Lipids	Soluble Proteins	Total Sugar
*Dictyota dichotoma*	3.07 ^a^ * ± 0.13 **	4.51 ^bc^ ± 0.005	57.87 ^a^ ± 0.04
*Sargassum muticum*	0.34 ^c^ ± 0.06	4.32 ^c^ ± 0.003	46.43 ^b^ ± 0.12
*Coralina officinalis*	0.16 ^d^ ± 0.01	4.71 ^b^ ± 0.004	34.93 ^d^ ± 0.11
*Ulva lactuca*	0.16 ^d^ ± 0.02	4.49 ^bc^ ± 0.002	24.53 ^e^ ± 0.04
*Cladophora laetevirens*	0.50 ^b^ ± 0.03	5.15 ^a^ ± 0.002	40.67 ^c^ ± 0.09
Mean	0.85	4.64	40.89
LSDp_0.05_	0.04	0.23	2.17

* The existence of identical letter indices in the averages (at least) indicates no statistically significant differences between them. Subsequent letter indices (a, b, c, d, e) define groups in descending order; ** Values are presented as means ± SD (n = 3).

**Table 4:** Total phenolic and flavonoid contents of the different extracts from the five sampled seaweeds.

Extracts		Total Phenolic Compound Content	Flavonoid Content
*Dictyota dichotoma*	MeOH	105.96 ± 2.37 ^c^	41.32 ± 0.52 ^c^
	EtOAc	189.33 ± 3.11 ^a^	98.45 ± 1.12 ^a^
	n-BuOH	116.07 ± 1.49 ^b^	48.04 ± 0.89 ^b^
*Sargassum muticum*	MeOH	130.53 ± 2.06 ^c^	115 ± 0.75 ^c^
	EtOAc	235.67 ± 1.13 ^a^	175.01 ± 0.87 ^a^
	n-BuOH	197.3 ± 2.70 ^b^	149 ± 1.67 ^b^
*Corallina officinalis*	MeOH	102.28 ± 4.78 ^c^	55.83 ± 0.21 ^c^
	EtOAc	211.04 ± 2.35 ^a^	75.41 ± 1.87 ^a^
	n-BuOH	158.37 ± 1.68 ^b^	57.07 ± 1.12 ^b^
*Ulva lactuca*	MeOH	110.53 ± 3.45 ^c^	70.35 ± 0.67 ^c^
	EtOAc	158.89 ± 2.79 ^a^	112.05 ± 1.89 ^a^
	n-BuOH	117.03 ± 0.75 ^b^	99 ± 0.31 ^b^
*Cladophora laetevirens*	MeOH	133.33 ± 2.90 ^c^	201.18 ± 0.73 ^c^
	EtOAc	215 ± 2.33 ^a^	331.05 ± 3.11 ^a^
	n-BuOH	187.67 ± 1.15 ^b^	286.29 ± 2.89 ^b^
Mean LSDp_0.05_		157.87	127.62
		2.34	1.24

TPC is expressed as μg gallic acid equivalents/mg of extract (μg GAE·mg^−1^). TFC is expressed as μg quercetin equivalents/mg of extract (μg QE·mg^−1^). Values are presented as means ± SD (n = 3). Different letters within a column (each seaweed) indicate significant differences with *p* < 0.05 using Tukey’s test.

**Table 5:** Antioxidant potentials of different fractions of the five seaweeds collected. NT: not tested.

Extracts		DPPH Assay IC_50_ (µg·mL^−1^)	ABTS Assay IC_50_ (µg·mL^−1^)	Reducing Power Assay A_0.5_ (µg·mL^−1^)	Phenanthroline Assay A_0.5_ (µg·mL^−1^)	SNP Assay A_0.5_ (µg·mL^−1^)
***Dictyota dichotoma***	MeOH	369.48 ± 1.28 ^b^	162.89 ± 2.46 ^a^	>200	190.28 ± 3.94 ^a^	112.20 ± 1.21 ^a^
	EtOAc	288.56 ± 2.98 ^c^	129.28 ± 1.78 ^c^	>200	85.71 ± 1.32 ^c^	109.87 ± 1.89 ^b^
	n-BuOH	327.89 ± 3.02 ^a^	142.56 ± 2.14 ^b^	>200	112.07 ± 2.11 ^b^	107.31 ± 2.11 ^c^
***Sargassum muticum***	MeOH	356.64 ± 2.20 ^a^	594.06 ± 1.15 ^a^	>200	170.28 ± 0.96 ^a^	62.50 ± 2.50 ^a^
	EtOAc	276.23 ± 1.98 ^c^	389.11 ± 2.13 ^c^	>200	72.07 ± 1.89 ^c^	45.79 ± 1.21 ^c^
	n-BuOH	347.74 ± 3.29 ^b^	499.07 ± 2.89 ^b^	>200	98.27 ± 1.11 ^b^	58.34 ± 1.78 ^b^
***Corallina officinalis***	MeOH	579.26 ± 1.39 ^a^	176.62 ± 2.10 ^a^	>200	107.28 ± 0.96 ^a^	36.46 ± 3.11 ^a^
	EtOAc	477.05 ± 2.94 ^b^	127.05 ± 1.89 ^c^	197.16 ± 2.43 ^a^	83.45 ± 0.88 ^c^	11.58 ± 0.79 ^c^
	n-BuOH	>800	169.03 ± 0.79 ^b^	>200	99.07 ± 1.06 ^b^	27.87 ± 1.11 ^b^
***Ulva lactuca***	MeOH	675.74 ± 3.66 ^a^	150.28 ± 1.15 ^a^	>200	26.61 ± 1.93 ^a^	118.24 ± 2.93 ^a^
	EtOAc	588.16 ± 3.22 ^b^	102.74 ± 1.89 ^c^	190.78 ± 3.84 ^a^	5.67 ± 0.82 ^c^	98.32 ± 1.87 ^c^
	n-BuOH	>800	136.01 ± 2.11 ^b^	>200	19.97 ± 0.97 ^b^	110.89 ± 2.87 ^b^
***Cladophora laetevirens***	MeOH	118.01 ± 1.12 ^b^	107.55 ± 1.62 ^a^	>200	28.98 ± 1.10 ^a^	63.71 ± 2.84 ^a^
	EtOAc	89.11 ± 0.98 ^c^	78.65 ± 0.96 ^c^	189.28 ± 2.94 ^a^	10.06 ± 0.88 ^c^	24.57 ± 1.03 ^c^
	n-BuOH	102.55 ± 1.29 ^a^	91.07 ± 1.12 ^b^	>200	23.50 ± 1.14 ^b^	43.79 ± 1.22 ^b^
BHA *		6.14 ± 0.41 ^e^	1.81 ± 0.10 ^e^	8.41 ± 0.67 ^b^	0.93 ± 0.07 ^e^	NT
BHT *		12.99 ± 0.41 ^f^	1.29 ± 0.30 ^f^	>200	2.24 ± 0.17 ^f^	NT
α-Tocopherol *		13.02 ± 5.17 ^g^	NT	34.93 ± 2.38 ^c^	NT	NT
Ascorbic acid *		NT	NT	6.77 ± 1.15 ^d^	NT	7.14 ± 0.05 ^d^
Trolox *		NT	NT	NT	NT	34.17 ± 1.23 ^e^
Mean		413.01	203.65	198.46	75.46	68.62
LSDp_0.05_		34.16	22.97	0.55	8.29	5.44

* Standard compounds. Note: Concentrations of positive controls BHA, BHT, α-Tocopherol, ascorbic acid, and trolox were 4 mg.mL^−1^. NT: not tested. IC_50_ and A_0.50_ were calculated by linear regression analysis and expressed as means ± SD (n = 3). The values with different superscripts (a, b, c, d, e, f, g) in the same columns are significantly different (*p* < 0.05). Values are presented as means ± SD (n = 3). Values are presented as means ± SD (n = 3).

**Table 6:** Descriptive statistics of antioxidant activity, TFC, and TFP.

Specification	*DPPH*	*ABTS*	*Reducing Power*	*Phenanthroline*	*SNP*	*TFC*	*TPC*
**Mean**	413.01	203.65	198.46	75.46	68.62	127.62	157.87
**Standard error**	34.16	22.97	0.55	8.29	5.44	12.71	6.56
**Median**	356.23	142.22	200.00	83.01	60.87	99.21	156.69
**Standard deviation**	229.16	154.07	3.67	55.58	36.50	85.29	43.99
**Kurtosis**	−0.95	1.33	4.51	−0.53	−1.54	0.55	−1.37
**Skewness**	0.29	1.65	−2.37	0.55	0.04	1.23	0.27
**Range**	711.87	517.52	13.66	189.37	110.38	293.30	139.23
**Minimum**	88.13	77.69	186.34	4.85	10.79	40.80	97.50
**Maximum**	800.00	595.21	200.00	194.22	121.17	334.10	236.73
**Coefficient of variation V (%)**	55.49	75.65	1.85	73.66	53.20	66.83	27.86

**Table 7:** Pearson’s simple correlation coefficients between the different antioxidant tests.

Specification	DPPH	ABTS	Reducing Power	Phenanthroline	SNP
**DPPH**	1.00				
**ABTS**	0.18	1.00			
**Reducing power**	0.36	0.30	1.00		
**Phenanthroline**	0.23	0.60	0.32	1.00	
**SNP**	0.39	0.07	0.38	0.21	1.00

**Table 8:** Pearson’s simple correlation coefficients between antioxidant activity and bioactive compounds.

Specifications	*DPPH*	*ABTS*	*Reducing Power*	*Phenanthroline*	*SNP*	*TFC*	*TPC*
**DPPH**	1.00						
**ABTS**	−0.05	1.00					
**Reducing power**	0.14	0.31	1.00				
**Phenanthroline**	0.02	0.52	0.45	1.00			
**SNP**	0.22	−0.12	0.19	0.04	1.00		
**TFC**	−0.70	−0.08	−0.41	−0.54	−0.41	1.00	
**TPC**	−0.43	0.13	−0.32	−0.28	−0.55	0.57	1.00

**Table 9:** Antimicrobial activity of seaweed species.

		Inhibition Diameter (mm)			
Seaweed	Bacteria	1000 µg·mL^−1^	500 µg·mL^−1^	250 µg·mL^−1^	125 µg·mL^−1^
*Dictyota dichotoma*	*E. coli*	26.00 ^a^ ± 1.14	19.50 ^a^ ± 0.71	15.50 ^a^ ± 0.71	13.50 ^a^ ± 0.71
	*S. aureus*	14.75 ^b^ ± 0.35	13.50 ^b^ ± 0.71	12.50 ^b^ ± 0.71	12.5 ^b^ ± 0.71
	Mean	20.4	16.50	14.0	12.5
	LSDp_0.05_	1.1	0.90	0.7	0.7
*Sargassum muticum*	*E. coli*	26.50 ^a^ ± 0.71	19.25 ^a^ ± 1.06	17.25 ^a^ ± 1.77	15.25 ^a^ ± 0.35
	*S. aureus*	14.25 ^b^ ± 0.35	13.00 ^b^ ± 1.41	11.25 ^b^ ± 1.06	9.75 ^b^ ± 1.06
	Mean	20.4	16.1	14.3	12.5
	LSDp_0.05_	1.1	0.9	0.8	0.7
*Corallina officinalis*	*E. coli*	22.50 ^a^ ± 0.71	18.50 ^a^ ± 0.71	16.25 ^a^ ± 1.06	11.50 ^a^ ± 0.71
	*S. aureus*	11.50 ^b^ ± 0.71	10.75 ^b^ ± 0.35	9.75 ^b^ ± 1.06	9.75 ^b^ ± 1.06
	Mean	17.0	14.6	13.0	10.6
	LSDp_0.05_	0.9	Ns	0.7	0.6
*Ulva lactuca*	*E. coli*	16.00 ^a^ ± 1.41	12.50 ^a^ ± 0.71	10.50 ^a^ ± 0.71	9.50 ^a^ ± 0.71
	*S. aureus*	13.50 ^b^ ± 0.71	11.50 ^b^ ± 0.71	10.50 ^a^ ± 0.71	9.00 ^a^ ± 1.41
	Mean	14.8	12.0	10.5	9.3
	LSDp_0.05_	0.8	0.7	ns	ns
*Cladophora laetevirens*	*E. coli*	15.75 ^a^ ± 1.06	13.00 ^a^ ± 1.41	11.50 ^a^ ± 0.71	8.50 ^a^ ± 0.71
	*S. aureus*	12.50 ^b^ ± 0.71	11.25 ^b^ ± 0.35	10.25 ^b^ ± 0.35	7.50 ^b^ ± 0.71
	Mean	14.1	12.1	10.9	8.0
	LSDp_0.05_	0.8	0.7	0.6	0.5

The existence of identical letter indices in the averages (at least) indicates no statistically significant differences between them. Subsequent letter indices (a, b, c, d, e) define groups in descending order; Values are presented as means ± SD (n = 3); ns—not significant at p_0.05_.

**Table 10:** Identification of the main morphological characteristics of the raw algae collected on the Algerian northeast coast.

Seaweed	Color	Size (cm)	Thalles Morphology
*Dictyota spiralis* (Kützing) Grunow	Dark brown and olive green	10 and 20	Thallus in the form of flattened, branched stems, with branches pointing in different directions. The fronds are divided into lobes or segments, giving it a feathery appearance.
*Sargassum muticum* (Yendo) Fensholt	Golden brown and slightly yellow	45 and 60	Branched stems (stolons) Leaves are toothed and lanceolate.
*Corallina officinalis* Linnaeus	Light red, slightly brown with dark pink areas	10 and 25	Thallus is composed of branched, calcified structures resembling small branches forming dense tufts
*Ulva lactuca* Linnaeus	Bright green	3 and 5	Its thallus consists of thin, small, flat, smooth and relatively translucent, ribbon-shaped green leaves.
*Cladophora laetevirens* (Dillwyn) Kützing	Slightly dark green	20 and 40	Its thallus consists of cylindrical branched filaments that form dense tufts.

## Data Availability

The data presented in this study are available for a limited time upon request from the corresponding author.

## Supplementary Materials

The following supporting information can be downloaded at: https://www.mdpi.com/article/10.3390/md22060273/s1, Table S1. Phenolic profile of *Dictyota dichotoma* determined by UPLC-ESI-MS-MS; Table S2. Phenolic profile of *Sargassum muticum* determined by UPLC-ESI-MS-MS; Table S3. Phenolic profile of *Coralina officinalis* determined by UPLC-ESI-MS-MS.; Table S4. Phenolic profile of *Ulva lactuca* determined by UPLC-ESI-MS-MS; Table S5. Phenolic profile of *Cladophora laetevirens* determined by UPLC-ESI-MS-MS; Table S6. Descriptive statistics of total phenolic and flavonoids content; Table S7. Descriptive statistics for different antioxidant assays, including DPPH, ABTS, of Reducing power, Phenanthroline, and SNP; Figure S1: Phenolic profile of *Dictyota dichotoma* determined by UPLC-ESI-MS-MS; Figure S2: Phenolic profile of *Sargassum muticum* determined by UPLC-ESI-MS-MS; Figure S3: Phenolic profile of *Coralina officinalis* determined by UPLC-ESI-MS-MS; Figure S4: Phenolic profile of *Ulva lactuca* determined by UPLC-ESI-MS-MS; Figure S5: Phenolic profile of *Cladophora laetevirens* determined by UPLC-ESI-MS-MS.
