# Supplementary material for: Exploring Bioactive Components and Assessing Antioxidant and Antibacterial Activities in Five Seaweed Extracts from the Northeastern Coast of Algeria
Source: Mar Drugs. 2024 Jun 12;22(6):273. doi: 10.3390/md22060273 (PMC11205126; doi:10.3390/md22060273)
Supplement: Supplementary file 1 [file marinedrugs-22-00273-s001.zip › marinedrugs-3021288-supplementary.pdf]

Table S1. Phenolic profile of *Dictyota dichotoma* determined by UPLC-ESI-MS-MS. Nd = "not detected": +/-: positive/negative; Rt: retention time; ESI: electrospray ionization; Voltage CE: capillary electrospray.

Name	Molecular Formula	Rt	Molecular Weight (g.mol^{-1})	Precursor ion m/z	Product ion m/z	ESI (+/-)	Voltage CE (v)	Max intensity			Area %		
								Dictyota dichotoma					
								MeOH	EtOAc	n-BuOH	MeOH	EtOAc	n-BuOH
**Kaempferol**	$C_{15}H_{10}O_6$	23.5	286.24	287.1	255.25	+	-8	8225	8225	7134	3.55	2.36	2.39
**Esculin**	$C_{15}H_{16}O_9$	30.45	340.28	341.3	177	+	-7	10273	5340	5209	4.43	1.53	1.75
**Hespertin**	$C_{16}H_{14}O_6$	37.7	302.28	300.9	255.25	-	10	880	8946	9767	0.38	2.56	3.28
**Vanillin**	$C_8H_8O_3$	48.1	152.15	153.1	71.15	+	-22	31203	9226	9216	**13.46**	2.64	3.09
**4-Hydroxy Coumarin Acid**	$C_9H_6O_3$	47.7	162.14	160.8	117.1	-	22	970	590	622	0.42	0.17	0.21
**Naringenin**	$C_{15}H_{12}O_5$	48.15	272.25	273.1	147.15	+	-25	1880	1646	1736	0.81	0.47	0.58
**Gallic acid**	$C_7H_6O_5$	18.2	170.12	168.8	125.1	-	16	Nd	123	Nd	Nd	0.04	Nd
**Chlorogenic Acid**	$C_{16}H_{18}O_9$	20.5	354.31	355	277.4	+	-10	Nd	435	496	Nd	0.12	0.17
**Benzoic Acid**	$C_7H_6O_2$	47.6	122.12	123.1	91.2	+	-12	Nd	55499	54617	Nd	**15.91**	**18.34**
**Quercetin**	$C_{15}H_{10}O_7$	47.85	302.23	303.1	85.05	+	-41	2490	2046	Nd	1.07	0.59	Nd
**Chrysin**	$C_{15}H_{10}O_4$	48	254.24	255.1	223.3	+	-8	121750	Nd	Nd	**52.52**	Nd	Nd
**Rutin**	$C_{27}H_{30}O_{16}$	41.5	610.517	611.2	73.2	+	-42	2360	Nd	Nd	1.02	Nd	Nd
**Caffeic Acid**	$C_9H_8O_4$	47.8	180.16	178.8	135.1	-	19	990	Nd	Nd	0.43	Nd	Nd

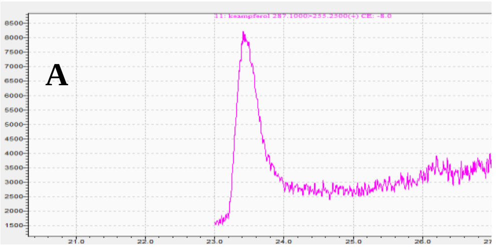

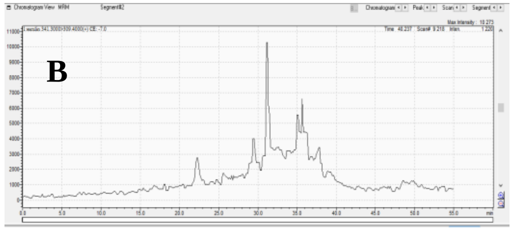

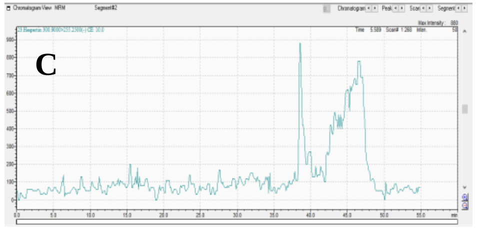

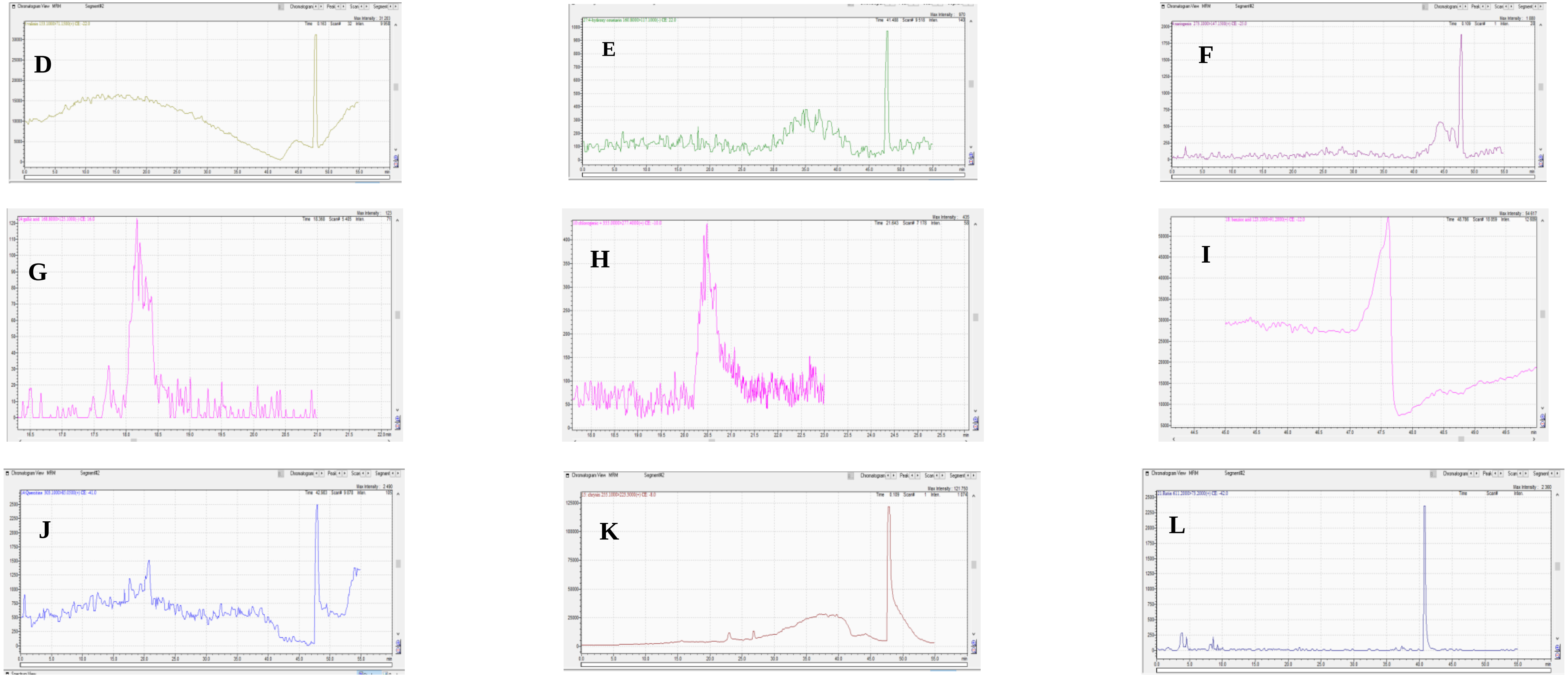
D
E
F
G
H
I
J
K
L

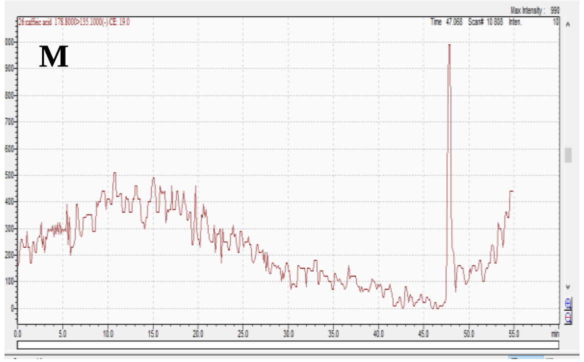

Figure S1: Phenolic profile of *Dictyota dichotoma* determined by UPLC-ESI-MS-MS. Nd = "not detected": +/- : positive/negative; Rt: retention time; ESI: electrospray ionization; Voltage CE: capillary electrospray; (**A**): kaempferol; (**B**): esculin; (**C**): hespertin; (**D**): vanillin; (**E**): 4-hydroxy coumarin acid; (**F**): naringenin; (**G**): gallic acid; (**H**): chlorogenic acid; (**I**): benzoic acid; (**J**): quercetin; (**K**): chrysin; (**L**): rutin; (**M**): caffeic acid.

Table S2. Phenolic profile of *Sargassum muticum* determined by UPLC-ESI-MS-MS. Nd = "not detected": +/- : positive/negative; Rt: retention time; ESI: electrospray ionization; Voltage CE: capillary electrospray.

Name	Molecular Formula	Rt	Molecular Weight (g.mol^{-1})	Precursor ion m/z	Product ion m/z	ESI (+/-)	Voltage CE (v)	Max intensity			Area %		
								Sargassum muticum					
								MeOH	EtOAc	n-BuOH	MeOH	EtOAc	n-BuOH
**Chlorogenic Acid**	$C_{16}H_{18}O_9$	20.45	354.31	355	277.4	+	-10	Nd	461	467	Nd	0.14	0.16
**Kaempferol**	$C_{15}H_{10}O_6$	23.4	286.24	287.1	255.25	+	-8	4280	8192	7114	1.87	2.41	2.36
**Esculin**	$C_{15}H_{16}O_9$	30.4	340.28	341.3	177	+	-7	9722	5431	5292	4.26	1.60	1.76
**Hespertin**	$C_{16}H_{14}O_6$	37.7	302.28	300.9	255.25	-	10	1000	9663	9966	0.44	2.84	3.31
**Vanillin**	$C_8H_8O_3$	48.05	152.15	153.1	71.15	+	-22	32354	8282	9626	**14.17**	2.44	3.20
**Benzoic Acid**	$C_7H_6O_2$	47.6	122.12	123.1	91.2	+	-12	Nd	53925	54795	Nd	**15.86**	**18.20**
**Naringenin**	$C_{15}H_{12}O_5$	47.8	272.25	273.1	147.15	+	-25	1880	1610	1936	0.82	0.47	0.64
**Quercetin**	$C_{15}H_{10}O_7$	48	302.23	303.1	85.05	+	-41	2500	1930	Nd	1.09	0.57	Nd
**Chrysin**	$C_{15}H_{10}O_4$	48	254.24	255.1	223.3	+	-8	122384	Nd	Nd	**53.60**	Nd	Nd
**Rutin**	$C_{27}H_{30}O_{16}$	41.5	610.517	611.2	73.2	+	-42	1550	Nd	Nd	0.68	Nd	Nd
**Caffeic Acid**	$C_9H_8O_4$	47.8	180.16	178.8	135.1	-	19	980	Nd	Nd	0.43	Nd	Nd
**4-Hydroxy Coumarin Acid**	$C_9H_6O_3$	47.7	162.14	160.8	117.1	-	22	910	Nd	520	0.40	Nd	0.17

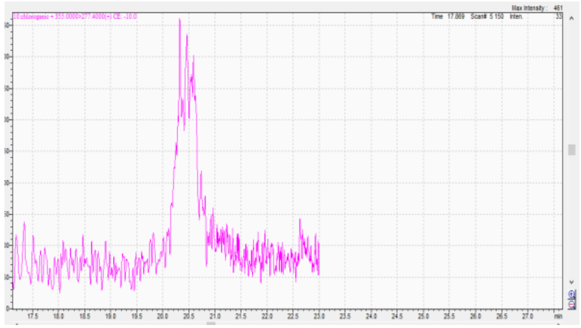

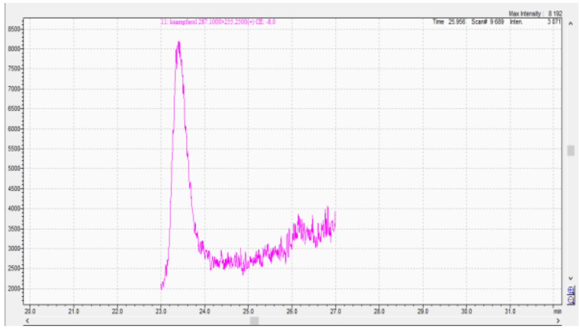

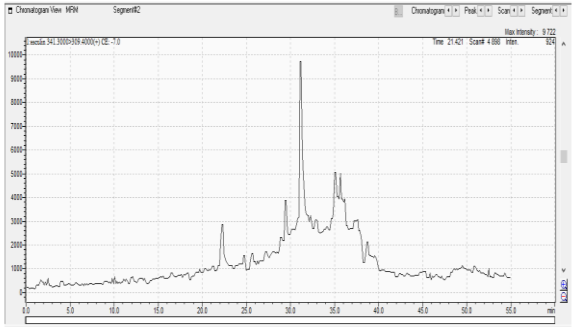

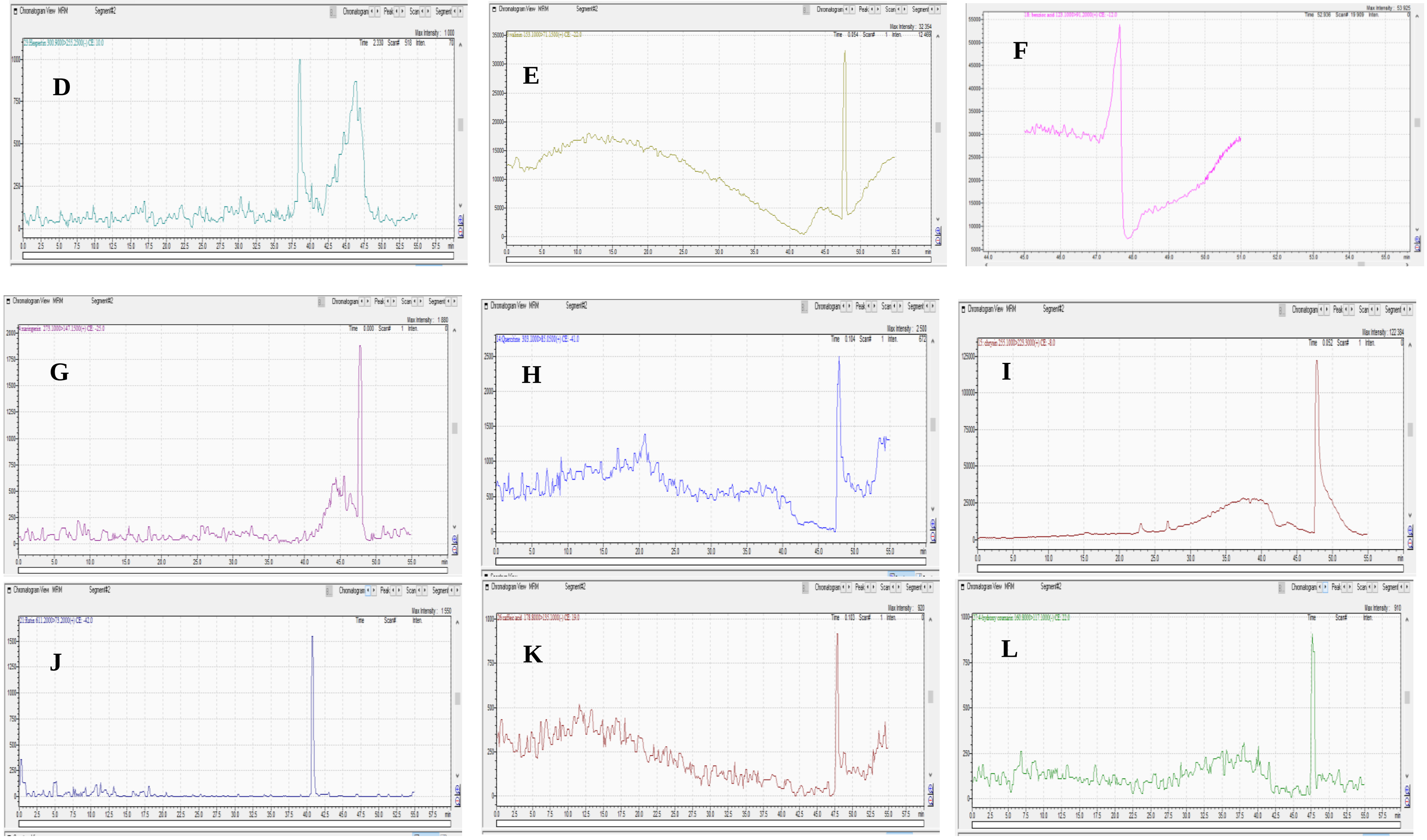
D
E
F
G
H
I
J
K
L

Figure S2: Phenolic profile of *Sargassum muticum* determined by UPLC-ESI-MS-MS. Nd = "not detected": +/- : positive/negative; Rt: retention time; ESI: electrospray ionization; Voltage CE: capillary electrospray; (**A**): chlorogenic acid; (**B**): kaempferol; (**C**): esculin; (**D**): hespertin; (**E**): vanillin; (**F**): benzoic acid; (**G**): naringenin; (**H**): quercetin; (**I**): chrysin; (**J**): rutin; (**K**): caffeic acid; (**L**): 4-hydroxy coumarin acid

Table S3. Phenolic profile of *Coralina officinalis* determined by UPLC-ESI-MS-MS. Nd = "not detected": +/- : positive/negative; Rt: retention time; ESI: electrospray ionization; Voltage CE: capillary electrospray.

**Name**	**Molecular Formula**	**Rt**	**Molecular Weight (g.mol^{-1})**	**Precursor ion m/z**	**Product ion m/z**	**ESI (+/-)**	**Voltage CE (v)**	**Max intensity**	**Area %**

								Coralina officinalis					
								MeOH	EtOAc	n-BuOH	MeOH	EtOAc	n-BuOH
**Chlorogenic Acid**	$C_{16}H_{18}O_9$	20.35	354.31	355	277.4	+	-10	Nd	503	504	Nd	0.17	0.17
**Kaempferol**	$C_{15}H_{10}O_6$	23.5	286.24	287.1	255.25	+	-8	Nd	7692	6853	Nd	2.64	2.32
**Esculin**	$C_{15}H_{16}O_9$	30.4	340.28	341.3	177	+	-7	7902	5285	5036	3.59	1.82	1.71
**Hespertin**	$C_{16}H_{14}O_6$	37.7	302.28	300.9	255.25	-	10	1020	10100	9416	0.46	3.47	3.19
**Vanillin**	$C_8H_8O_3$	48.1	152.15	153.1	71.15	+	-22	30221	8842	9698	**13.72**	3.04	3.28
**Benzoic Acid**	$C_7H_6O_2$	47.6	122.12	123.1	91.2	+	-12	Nd	51043	54657	Nd	**17.55**	**18.51**
**Naringenin**	$C_{15}H_{12}O_5$	47.75	272.25	273.1	147.15	+	-25	1780	1664	1854	0.81	0.57	0.63
**Quercetin**	$C_{15}H_{10}O_7$	47.9	302.23	303.1	85.05	+	-41	2610	1922	1924	1.18	0.66	0.65
**4-Hydroxy Coumarin Acid**	$C_9H_6O_3$	47.7	162.14	160.8	117.1	-	22	650	614	498	0.30	0.21	0.17
**Chrysin**	$C_{15}H_{10}O_4$	48	254.24	255.1	223.3	+	-8	122334	Nd	Nd	**55.53**	Nd	Nd
**Rutin**	$C_{27}H_{30}O_{16}$	41.5	610.517	611.2	73.2	+	-42	1310	Nd	Nd	0.59	Nd	Nd
**Caffeic Acid**	$C_9H_8O_4$	47.8	180.16	178.8	135.1	-	19	870	Nd	Nd	0.39	Nd	Nd

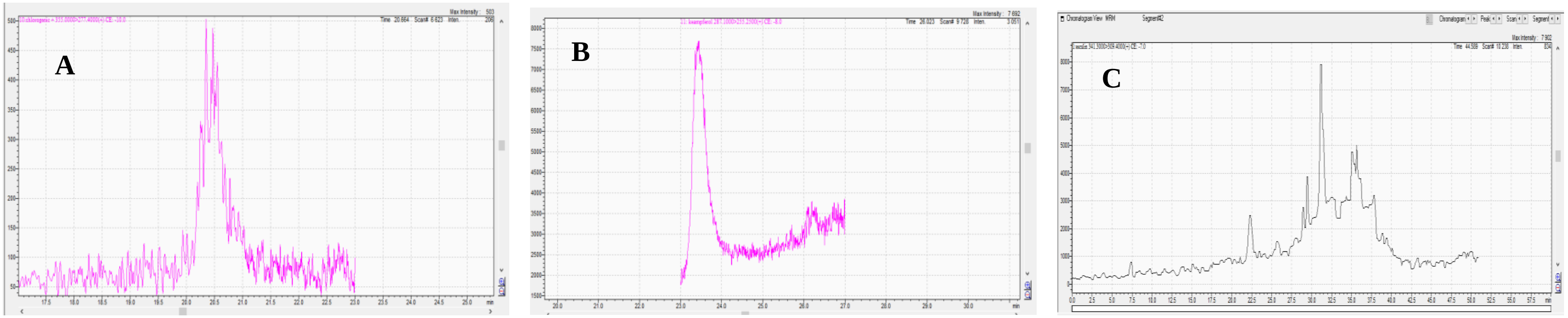

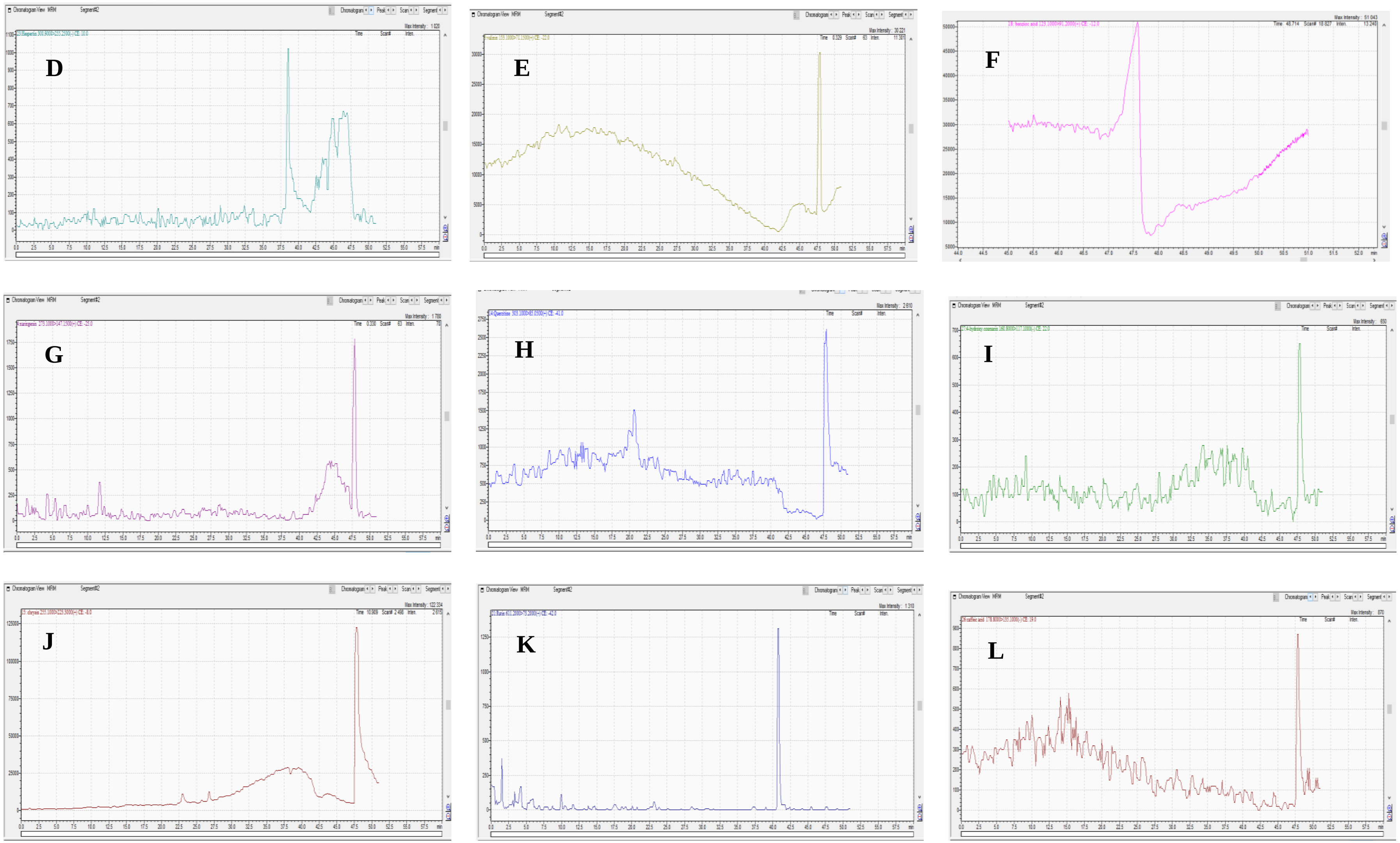
D
E
F
G
H
I
J
K
L

Figure S3: Phenolic profile of *Coralina officinalis* determined by UPLC-ESI-MS-MS. Nd = "not detected": +/- : positive/negative; Rt: retention time; ESI: electrospray ionization; Voltage CE: capillary electrospray; (**A**): chlorogenic acid; (**B**): kaempferol; (**C**): esculin; (**D**): hespertin; (**E**): vanillin; (**F**): benzoic acid; (**G**): naringenin; (**H**): quercetin; (**I**): 4-hydroxy coumarin acid; (**J**): chrysin; (**K**): rutin; (**L**): caffeic acid.

Table S4. Phenolic profile of *Ulva lactuca* determined by UPLC-ESI-MS-MS. Nd = "not detected": +/- : positive/negative; Rt: retention time; ESI: electrospray ionization; Voltage CE: capillary electrospray.

**Name**	**Molecular Formula**	**Rt**	**Molecular Weight (g.mol^{-1})**	**Precursor ion m/z**	**Product ion m/z**	**ESI (+/-)**	**Voltage CE (v)**	**Max intensity**			**Area %**		
								Ulva lactuca					
								MeOH	*EtOAc*	*n-BuOH*	*MeOH*	*EtOAc*	*n-BuOH*
**Kaempferol**	$C_{15}H_{10}O_6$	23.5	286.24	287.1	255.25	+	-8	1060	7700	6651	0.62	2.49	2.41
**Esculin**	$C_{15}H_{16}O_9$	30.45	340.28	341.3	177	+	-7	16206	5382	5234	9.51	1.74	1.89
**Vanillin**	$C_8H_8O_3$	48.1	152.15	153.1	71.15	+	-22	67034	8532	9991	**39.32**	2.76	3.62
***P*-coumaric Acid**	$C_9H_8O_3$	41.8	164.0473	165.1	59.1	+	-17	980	Nd	Nd	0.57	Nd	Nd
**Naringenin**	$C_{15}H_{12}O_5$	48.15	272.25	273.1	147.15	+	-25	3130	1806	1794	1.84	0.58	0.65

**Quercetin**	$C_{15}H_{10}O_7$	47.85	302.23	303.1	85.05	+	-41	4721	1994	1932	2.77	0.64	0.70
**Chrysin**	$C_{15}H_{10}O_4$	48	254.24	255.1	223.3	+	-8	2314	Nd	Nd	1.36	Nd	Nd
**Rutin**	$C_{27}H_{30}O_{16}$	41.5	610.517	611.2	73.2	+	-42	4881	Nd	Nd	2.86	Nd	Nd
**Myricetin**	$C_{15}H_{10}O_8$	41	318.2351	336.25	46.15	+	-20	1020	Nd	Nd	0.60	Nd	Nd
**Chlorogenic Acid**	$C_{16}H_{18}O_9$	6.8	354.31	355	73.15	+	-34	1480	495	523	0.87	0.16	0.19
**Hespertin**	$C_{16}H_{14}O_6$	37.7	302.28	300.9	255.25	-	10	Nd	10644	9475	Nd	3.44	3.43
**Benzoic Acid**	$C_7H_6O_2$	47.6	122.12	123.1	91.2	+	-12	Nd	51160	53248	Nd	**16.54**	**19.27**
**4-Hydroxy Coumarin Acid**	$C_9H_6O_3$	47.8	162.14	160.8	117.1	-	22	Nd	490	494	Nd	0.16	0.18

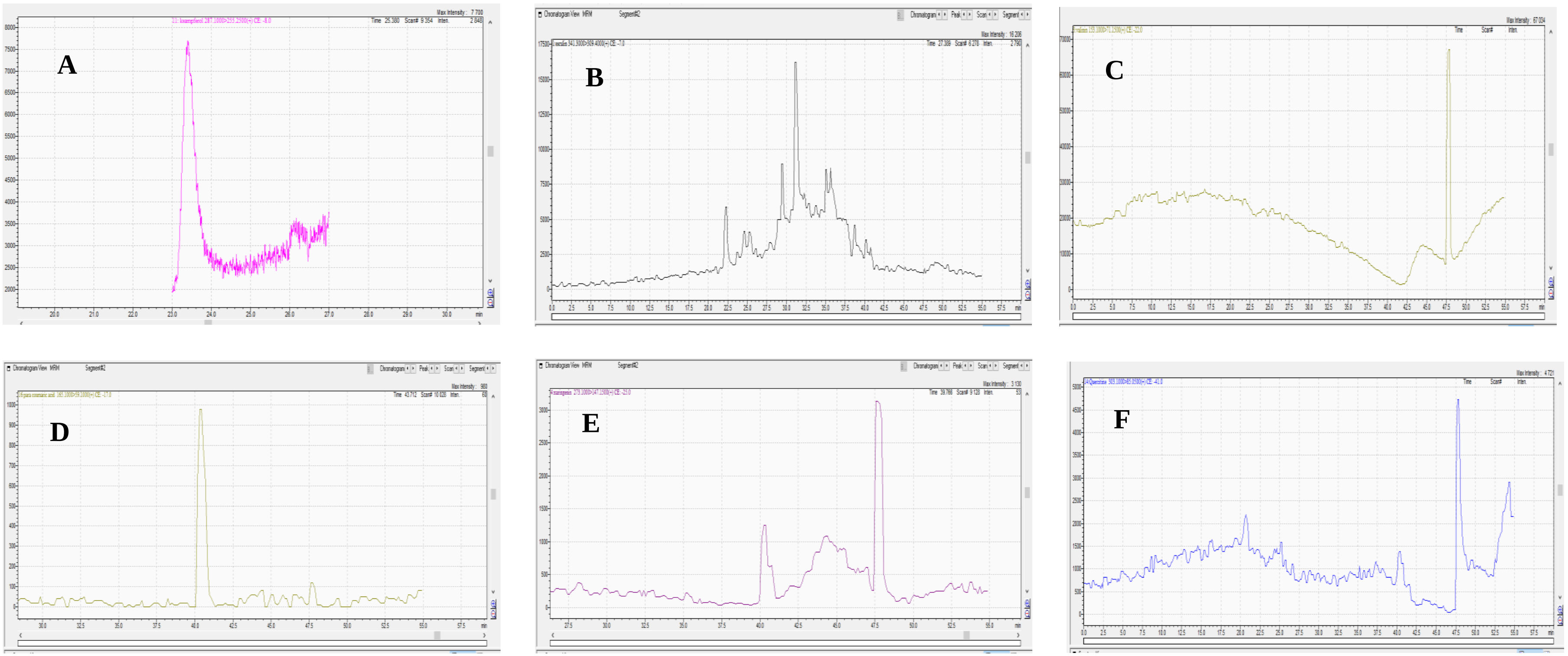

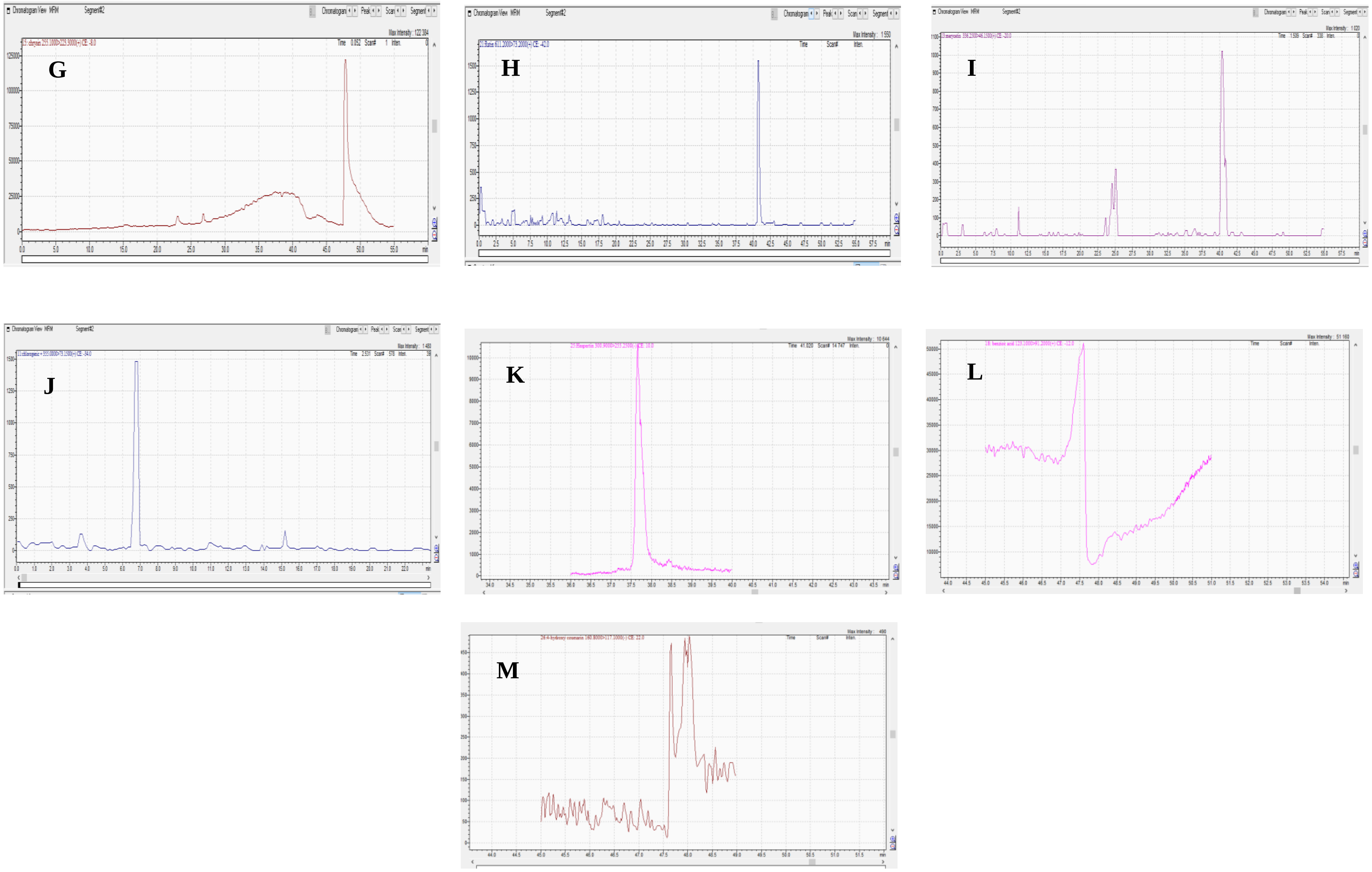
G
H
I
J
K
L
M

Figure S4: Phenolic profile of *Ulva lactuca* determined by UPLC-ESI-MS-MS. Nd = "not detected": +/- : positive/negative; Rt: retention time; ESI: electrospray ionization; Voltage CE: capillary electrospray; (**A**): kaempferol; (**B**): esculin; (**C**): vanillin; (**D**): P-cumaric acid; (**E**): naringenin; (**F**): quercetin; (**G**): chrysin; (**H**): rutin; (**I**): myricetin; (**J**): chlorogenic acid; (**K**): hespertin; (**L**): benzoic acid; (**M**): 4-hydroxy coumarin acid.

Table S5. Phenolic profile of *Cladophora laetevirens* determined by UPLC-ESI-MS-MS. Nd = "not detected": +/- : positive/negative; Rt: retention time; ESI: electrospray ionization; Voltage CE: capillary electrospray.

**Name**	**Molecular Formula**	**Rt**	**Molecular Weight (g.mol^{-1})**	**Precursor ion m/z**	**Product ion m/z**	**ESI (+/-)**	**Voltage CE (v)**	**Max intensity**			**Area %**		
								Cladophora laetevirens					
								MeOH	EtOAc	n-BuOH	MeOH	EtOAc	n-BuOH
**Naringenin**	$C_{15}H_{12}O_5$	48.15	272.25	273.1	147.15	+	-25	750	1772	1922	0.68	0.58	0.71
**Quercetin**	$C_{15}H_{10}O_7$	47.85	302.23	303.1	85.05	+	-41	1840	1992	1874	1.66	0.65	0.69
**Rutin**	$C_{27}H_{30}O_{16}$	41.5	610.517	611.2	73.2	+	-42	1650	Nd	Nd	1.49	Nd	Nd
**Cinnamic Acid**	$C_9H_8O_2$	9.5	148.1586	149.1	103.2	+	-23	360	Nd	Nd	0.33	Nd	Nd
**Hespertin**	$C_{16}H_{14}O_6$	37.7	302.28	300.9	255.25	-	10	4350	10985	10256	3.93	3.59	3.79
**Caffeic Acid**	$C_9H_8O_4$	47.8	180.16	178.8	135.1	-	19	1710	Nd	Nd	1.55	Nd	Nd
**4-Hydroxy Coumarin Acid**	$C_9H_6O_3$	48	162.14	160.8	117.1	-	22	1000	548	580	0.90	0.18	0.21
**Chlorogenic Acid**	$C_{16}H_{18}O_9$	20.55	354.31	355	277.4	+	-10	Nd	491	509	Nd	0.16	0.19
**Kaempferol**	$C_{15}H_{10}O_6$	23.5	286.24	287.1	255.25	+	-8	Nd	7739	6240	Nd	2.53	2.31
**Esculin**	$C_{15}H_{16}O_9$	30.4	340.28	341.3	309.4	+	-7	Nd	5096	5708	Nd	1.67	2.11
**Vanillin**	$C_8H_8O_3$	48.03	152.15	153.1	71.15	+	-22	Nd	8970	10623	Nd	2.93	3.93
**Benzoic Acid**	$C_7H_6O_2$	47.6	122.12	123.1	91.2	+	-12	Nd	51701	52437	Nd	**16.89**	**19.40**

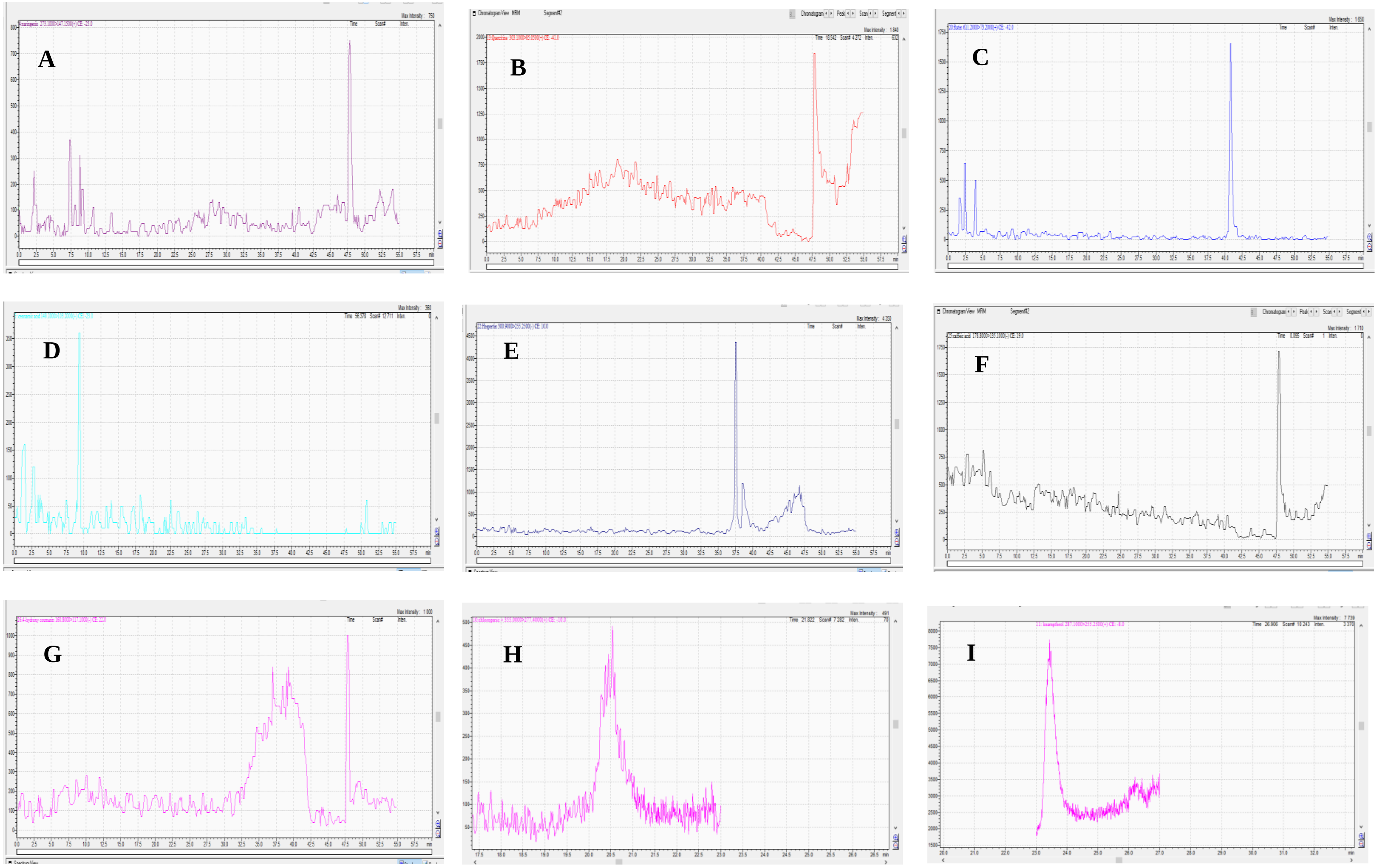
A
B
C
D
E
F
G
H
I

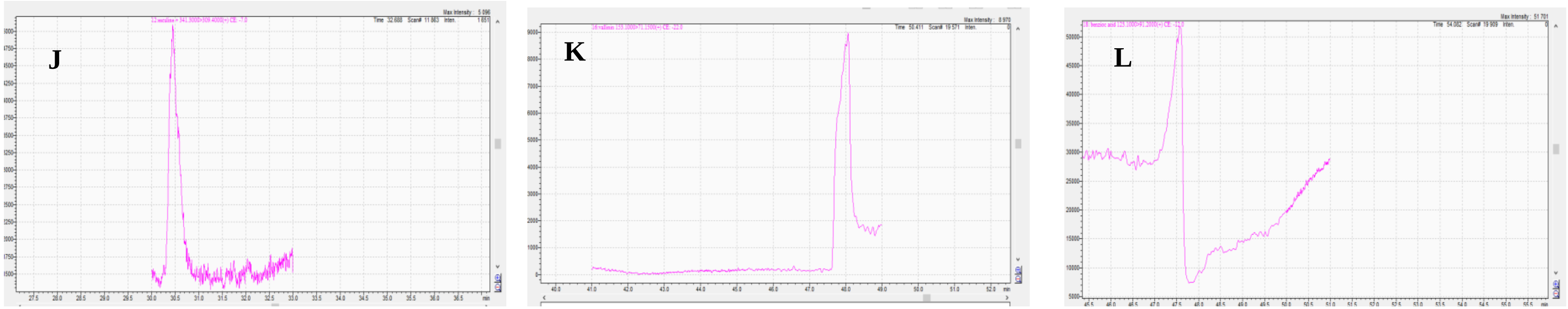

Figure S5: Phenolic profile of *Cladophora laetevirens* determined by UPLC-ESI-MS-MS. Nd = "not detected": +/- : positive/negative; Rt: retention time; ESI: electrospray ionization; Voltage CE: capillary electrospray; (**A**): naringenin; (**B**): quercetin; (**C**): rutin; (**D**): cinnamic acid; (**E**): hespertin; (**F**): caffeic acid; (**G**): 4-hydroxy coumarin acid; (**H**): chlorogenic acid; (**I**): kaempferol; (**J**): esculin; (**K**): vanillin; (**L**): benzoic acid.

Table S6. Descriptive statistics of total phenolic and flavonoid contents

Specification	TPC	TFC
**Mean**	157,87	127,62
**Standard error**	6,56	12,71
**Median**	156,69	99,21
**Standard deviation**	43,99	85,29
**Kurtosis**	-1,37	0,55
**Skewness**	0,27	1,23
**Range**	139,23	293,30
**Minimum**	97,50	40,80
**Maximum**	236,73	334,10
**Coefficient of variation V (%)**	27,86	66,83

Table S7. Descriptive statistics for different antioxidant assays, including DPPH, ABTS, reducing power, phenanthroline, and SNP.

Specification	DPPH	ABTS	Reducing power	Phenanthroline	SNP
**Mean**	365,52	179,87	187,37	66,77	62,97
**Standard error**	35,28	22,23	6,35	8,04	5,31
**Median**	347,02	136,03	200,00	72,03	58,01
**Standard deviation**	251,92	158,79	45,32	57,42	37,93
**Kurtosis**	-0,97	1,49	13,27	-0,51	-1,46
**Skewness**	0,27	1,53	-3,83	0,64	0,19
**Range**	794,27	594,22	192,26	193,36	114,08
**Minimum**	5,73	0,99	7,74	0,86	7,09
**Maximum**	800,00	595,21	200,00	194,22	121,17
**Coefficient of variation V (%)**	68,92	88,28	24,19	85,99	60,22
